# MFG-E8 promotes osteogenic transdifferentiation of smooth muscle cells and vascular calcification by regulating TGF-β1 signaling

**DOI:** 10.1038/s42003-022-03313-z

**Published:** 2022-04-19

**Authors:** Hou-Yu Chiang, Pao-Hsien Chu, Shao-Chi Chen, Ting-Hein Lee

**Affiliations:** 1 Department of Anatomy, College of Medicine, Chang Gung University, Taoyuan, Taiwan; 2 Graduate Institute of Biomedical Sciences, College of Medicine, Chang Gung University, Taoyuan, Taiwan; 3 Division of Cardiology, Department of Internal Medicine, Chang Gung Memorial Hospital, Linkou, Taiwan; 4 College of Medicine, Chang Gung University, Taoyuan, Taiwan; 5 Institute of Stem Cell and Translational Cancer Research, Chang Gung Memorial Hospital, Linkou, Taiwan

**Keywords:** Mechanisms of disease, Calcification

## Abstract

Vascular calcification occurs in arterial aging, atherosclerosis, diabetes mellitus, and chronic kidney disease. Transforming growth factor-β1 (TGF-β1) is a key modulator driving the osteogenic transdifferentiation of vascular smooth muscle cells (VSMCs), leading to vascular calcification. We hypothesize that milk fat globule–epidermal growth factor 8 (MFG-E8), a glycoprotein expressed in VSMCs, promotes the osteogenic transdifferentiation of VSMCs through the activation of TGF-β1-mediated signaling. We observe that the genetic deletion of MFG-E8 prevents calcium chloride-induced vascular calcification in common carotid arteries (CCAs). The exogenous application of MFG-E8 to aged CCAs promotes arterial wall calcification. MFG-E8-deficient cultured VSMCs exhibit decreased biomineralization and phenotypic transformation to osteoblast-like cells in response to osteogenic medium. MFG-E8 promotes β1 integrin–dependent MMP2 expression, causing TGF-β1 activation and subsequent VSMC osteogenic transdifferentiation and biomineralization. Thus, the established molecular link between MFG-E8 and vascular calcification suggests that MFG-E8 can be therapeutically targeted to mitigate vascular calcification.

## Abstract (web summary)

A molecular link between the milk fat globule–epidermal growth factor 8 (MFG-E8), activation of vascular calcification driver TGF-β1 and osteogenic differentiation of vascular smooth muscle cells suggests that MFG-E8 could be a therapeutic target for vascular calcification.

## Introduction

Vascular calcification is a pathological process associated with aging, hypertension, diabetes mellitus, and chronic kidney disease (CKD)^1–3^. Ectopic deposition of calcium phosphate minerals alters the biomechanical properties of blood vessels, resulting in reduced vessel elasticity and compliance and leading to increased cardiovascular morbidity and mortality^4^. Vascular calcification was once regarded as a degenerative disorder with passive precipitation of calcium phosphate in dead and dying cells; however, accumulating evidence indicates that vascular calcification is an active vascular smooth muscle cell (VSMC)–driven process that shares many features with embryonic bone formation^5^. Upon osteogenic stimuli such as high concentrations of extracellular phosphate and calcium, oxidized lipoproteins, reactive oxygen species, or inflammatory cytokines, VSMCs undergo a phenotypic switch and acquire osteoblast-like characteristics through the upregulation of osteogenic genes, such as runt-related transcription factor 2 (Runx2), msh homeobox 2, and osterix and through the downregulation of the expressions of VSMC differentiation marker genes, including smooth muscle α-actin (SMA) and smooth muscle myosin (SM-Myosin)^6,7^.

Transforming growth factor-β1 (TGF-β1) is a well-documented key modulator involved in vascular calcification. TGF-β1^7^, which is expressed in the endothelium, VSMCs, myofibroblasts, and infiltrated leukocytes in the arterial walls^8^, regulates the extracellular matrix (ECM) deposition and osteogenic transdifferentiation of VSMCs^9,10^. TGF-β is secreted as an inactive complex together with a latency-associated peptide (LAP). This complex interacts with the latent TGF-β binding proteins (LTBPs) and is anchored in the ECM^10,11^. TGF-β can be liberated from the complex, a phenomenon referred to as TGF-β activation, by the proteolytic cleavage of LAP, LTBP, or ECM proteins with several proteases including plasmin, matrix metalloproteinase 2 (MMP2), and MMP9^12^. Active TGF-β1 induces the osteoinductive signaling pathways by binding to a transmembrane receptor and initiates the phosphorylation of regulatory Smad2/3 protein, leading to the transcription of several osteogenic genes^13,14^. Studies have revealed that upregulation of MMPs in diseased vasculature leads to arterial calcification^15,16^.

Milk fat globule–epidermal growth factor 8 (MFG-E8) is a secreted, integrin-binding glycoprotein that contains multifunctional domains^17^. MFG-E8 is expressed by endothelial cells, VSMCs, and macrophages in aged and diseased vessels^18–20^. MFG-E8 has been identified as a novel biomarker of arterial aging^20,21^, and our recent work established the molecular link of MFG-E8 with the proinflammatory profiles of aging arterial walls and VSMCs^22^. A positive correlation between the serum MFG-E8 level and pulse wave velocity, an indicator of arterial stiffness, has been observed in older patients with type 2 diabetes mellitus or CKD^23,24^, suggesting the possible involvement of MFG-E8 in the pathogenesis of vascular calcification, which is one of the contributors for arterial stiffness. Recent work reported that MFG-E8-knockout (KO) mice exhibited impaired osteoblast differentiation through the attenuation of the expression of osteoblast genes, including Runx2^25^. Furthermore, MFG-E8 promotes the secretion of MMP2, a potent activator of TGF-β1, in macrophages and cancer cells^26,27^.

In this study, we hypothesize that MFG-E8 promotes VSMC osteogenic transdifferentiation and biomineralization through the activation of procalcifying TGF-β1 signaling. To test our hypothesis, two animal models of vascular calcification were applied to common carotid arteries (CCAs). We introduced MFG-E8 to aged CCAs to determine whether exogenous MFG-E8 can induce vascular calcification. Additionally, we subjected wild-type (WT) and MFG-E8-KO mice to a model of vascular calcification induced using periadventitial calcium chloride (CaCl_2_), which results in local CCA calcification. We also established an in vitro model of VSMC calcification with high extracellular calcium and phosphate concentrations^28,29^, both of which stimulate VSMC phenotypic transformation^28,30^. Our results indicated that MFG-E8 expression is significantly upregulated in calcifying arteries and that genetic deletion of MFG-E8 protects the calcifying vessels from pathological calcium deposition in the arterial walls. The treatment of CCAs with recombinant MFG-E8 induced vascular calcification. Moreover, we identified a novel role for MFG-E8 in promoting the osteogenic transdifferentiation of VSMCs through the activation of MMP-2-dependent TGF-β1-Smad2/3 signaling under osteogenic stimuli.

## Results

### Osteogenic stimuli induce the expression of MFG-E8 in calcifying VSMCs in vivo and in vitro

**Fig. 1:**
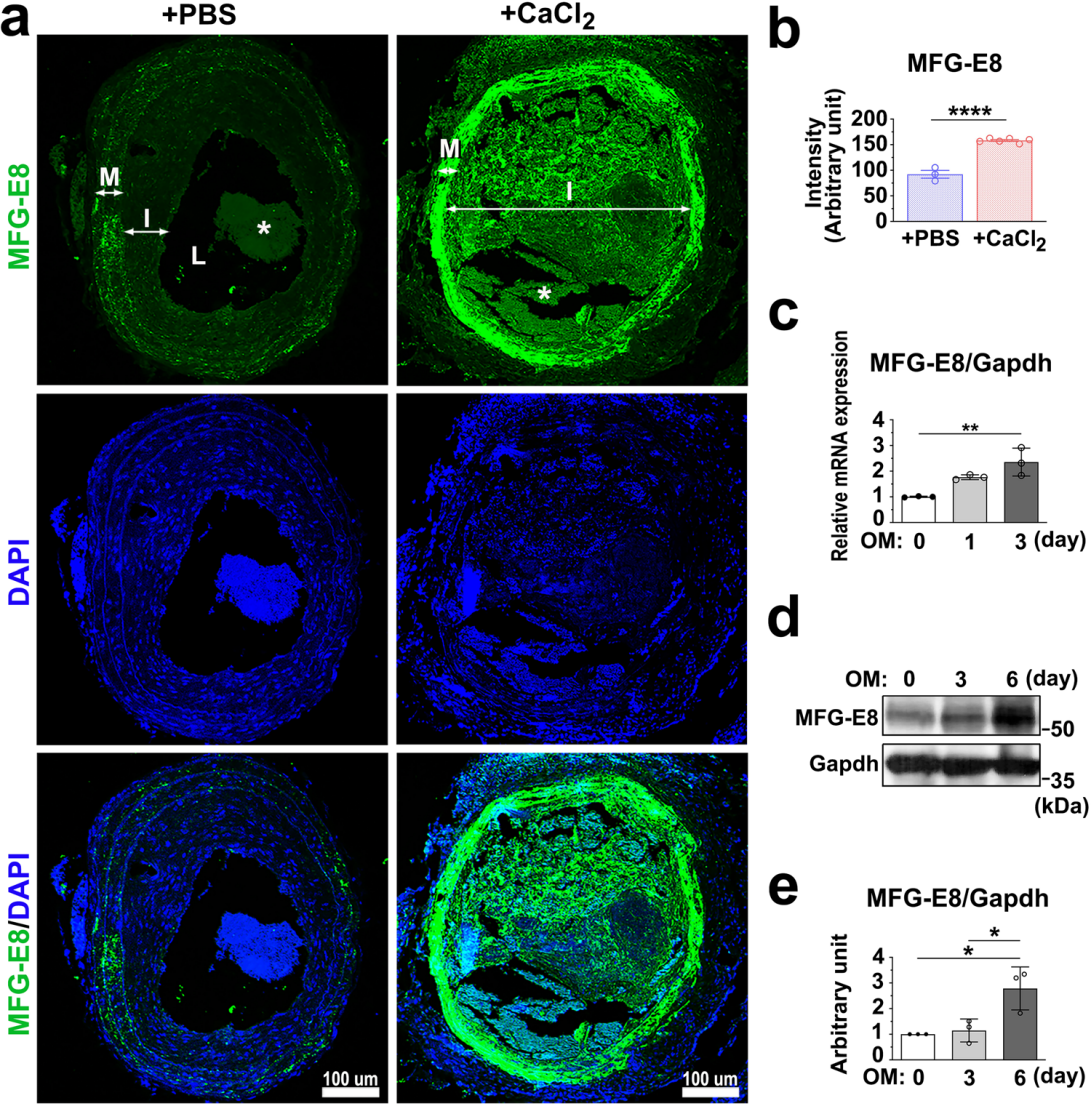
Milk fat globule–epidermal growth factor 8 (MFG-E8) expression is upregulated in calcifying vessels and vascular smooth muscle cells (VSMCs). **a**, **b** Common carotid arteries (CCAs) of wild-type mice were ligated, and phosphate-buffered saline (PBS) or CaCl_2_ (0.4 M) was applied on the vessels by using pluronic gel. **a** Representative immunofluorescence photographs display the expression of MFG-E8 in the ligated CCAs treated with PBS or CaCl_2_ at 21 days postligation. The neointima (I), media (M), and lumen (L) of the vessels are indicated. Blood clots in the lumen are labeled as “*”. Scale bar: 100 μm. **b** Quantification of fluorescence intensities of MFG-E8 in the arterial walls (PBS: *n*_mice_ = 3, CaCl_2,_ *n*_mice_ = 6). Results are presented as mean ± standard error of the mean. Each data point was derived from an assessment of three sections from one individual animal. ****P < 0.0001, obtained using a *t* test. **c**–**e** A10 VSMCs were cultured in osteogenic medium (OM) for 6 days. **c** The transcript expression of MFG-E8 was evaluated through quantitative real-time polymerase chain reaction (*n* = 3). Data are presented as mean ± standard deviation (SD). Three independent experiments were performed. Each data point is derived from each of the three repeated experiments. **P < 0.01, obtained using one-way analysis of variance (ANOVA) followed by Tukey’s multiple comparison test. **d** The protein expression of MFG-E8 was evaluated through western blotting. **e** Quantitative analyses of MFG-E8 levels normalized to those of glyceraldehyde 3-phosphate dehydrogenase (GAPDH) were conducted (*n* = 3). Data are presented as mean ± SD. Three independent experiments were performed. Each data point is derived from each of the three repeated experiments. *P < 0.05, as obtained using one-way ANOVA followed by Tukey’s multiple comparisons test.

VSMCs reportedly switch from the contractile to synthetic phenotype in ligation-induced vascular remodeling^31^. Synthetic VSMCs are pluripotent-like mesenchymal cells, and they transdifferentiate to osteoblast-like cells upon appropriate stimuli^32,33^. We first examined whether MFG-E8 is induced in the arterial wall upon osteogenic stimuli. We ligated the CCAs of WT mice and periadventitially applied CaCl_2_ onto ligated CCAs to prompt arterial calcification and the switch of VSMCs into the procalcifying phenotype. As depicted in Fig. 1a, b, the treatment of CaCl_2_ on ligated CCAs significantly increased the endogenous level of MFG-E8 in the neointima and media relative to that of phosphate-buffered saline (PBS). Because sex differences in vascular stiffness have been reported^34^, we subsequently analyzed these differences. As illustrated in Supplementary Fig. 1a, no sex-dependent difference in CaCl_2_-induced MFG-E8 expression was observed. To determine whether VSMCs are the main cells driving vascular calcification, we cultured VSMCs in an osteogenic medium (OM) containing high calcium and phosphate concentrations. Consistent with the in vivo results, the mRNA and protein expression levels of MFG-E8 were remarkably elevated in A10 VSMCs cultured in OM in a time-dependent manner (Fig. 1c–e). These results suggest that MFG-E8 upregulation may be responsible for inducing VSMC calcification.

### MFG-E8 plays an essential role in the development of vascular calcification

**Fig. 2:**
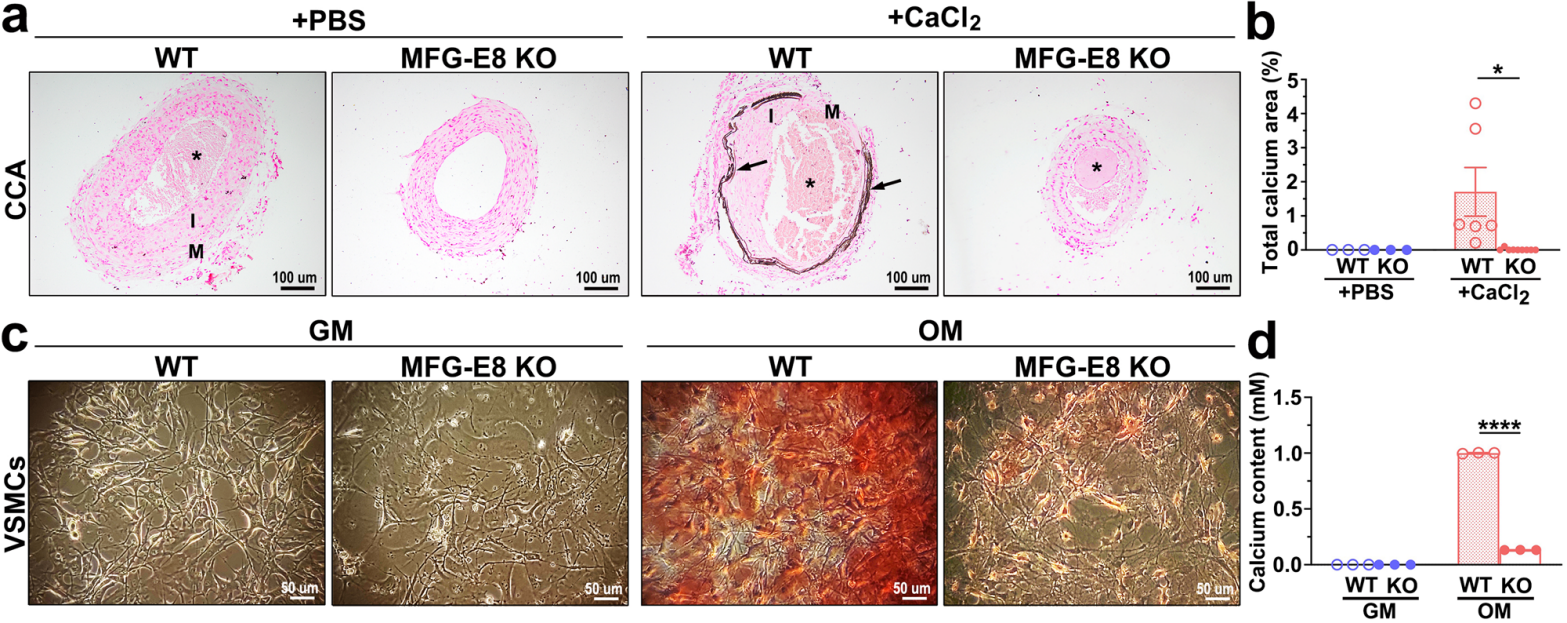
Milk fat globule–epidermal growth factor 8 (MFG-E8) deficiency attenuates calcium deposition in calcifying vessels and vascular smooth muscle cells (VSMCs). **a**, **b** Ligated common carotid arteries (CCAs) of wild-type (WT) and MFG-E8-knockout (KO) mice were treated with pluronic gel containing 0.4 M CaCl_2_ to induce vascular calcification. **a** Representative images of von Kossa stain on CCAs sections from four groups of mice 21 days postligation. Arrows indicate calcium deposit in the vascular walls. The neointima (I), media (M), and blood clots (*) are indicated. Scale bar: 100 μm. **b** Quantification of calcified area in the arterial walls (WT + phosphate-buffered saline (PBS): *n*_mice_ = 3, KO + PBS: *n*_mice_ = 3, WT + CaCl_2_: *n*_mice_ = 6, KO + CaCl_2_: *n*_mice_ = 9). Results are presented as mean ± standard error of the mean. Each data point is derived from an assessment of three sections of one individual animal. *P < 0.05, obtained using one-way analysis of variance (ANOVA) followed by Tukey’s multiple comparison test. **c**, **d** VSMCs derived from the aortas of WT and MFG-E8-KO mice were cultured in growth medium (GM) and osteogenic medium (OM) for 7 days. **c** Representative microscopy images of alizarin red stain (ARS) of 7-day cultured VSMCs. Scale bar: 50 μm. **d** Quantitative analysis of ARS contents in VSMCs (*n* = 3). Data are presented as mean ± standard deviation. Three independent experiments were performed, and each experiment was repeated with similar results. The data were derived from one of the representative experiments. ****P < 0.0001, as obtained using one-way ANOVA followed by Tukey’s multiple comparisons test.

To further elucidate whether MFG-E8 is instrumental in the pathogenesis of vascular calcification, the CCAs of WT and MFG-E8-KO mice were subjected to an animal model of vascular calcification induced by CaCl_2_, as previously described. Administration of CaCl_2_ onto the ligated CCAs of WT mice significantly increased calcium deposition in the arterial walls compared with the effect of PBS (Fig. 2a, b). By contrast, genetic deletion of MFG-E8 reduced the CaCl_2_-induced increase in arterial calcium content (Fig. 2a, b). No sex-dependent differences in CaCl_2_-induced vascular calcification were observed in the WT and KO mice (Supplementary Fig. 1b). We subsequently assessed the extent of calcification in VSMCs derived from WT and KO aortas in an in vitro biomineralization model. Alizarin red staining of 7-day-cultured primary VSMCs revealed remarkable calcium deposition in WT but not in MFG-E8-KO VSMCs (Fig. 2c, d). To verify that the attenuated accumulation of calcium in MFG-E8-KO VSMCs was not attributable to decreased cell growth and increased apoptosis, as compared with WT cells, immunoblotting of proliferating cell nuclear antigen (PCNA) and caspase 3 was conducted to evaluate the cell growth and apoptosis of WT and KO VSMCs, respectively. Because MFG-E8 has been verified to promote VSMC proliferation^21^, as expected, primary VSMCs with MFG-E8 deficiency exhibited decreased expression of PCNA compared with WT VSMCs cultured in growth medium (GM), as presented in Supplementary Fig. 2a, b. No difference in apoptosis was noted between WT and MFG-E8-KO VSMCs cultured in GM, as indicated through the immunoblotting of caspase 3 (Supplementary Fig. 2c, d). By contrast, culturing cells in OM did not elicit any significant difference in PCNA and caspase 3 expression between WT and KO VSMCs, indicating no difference in the growth and apoptosis of WT and MFG-E8-KO VSMCs cultured in OM. Together, these results indicated that MFG-E8 deletion ameliorated VSMC mineralization in vivo and in vitro.

**Fig. 3:**
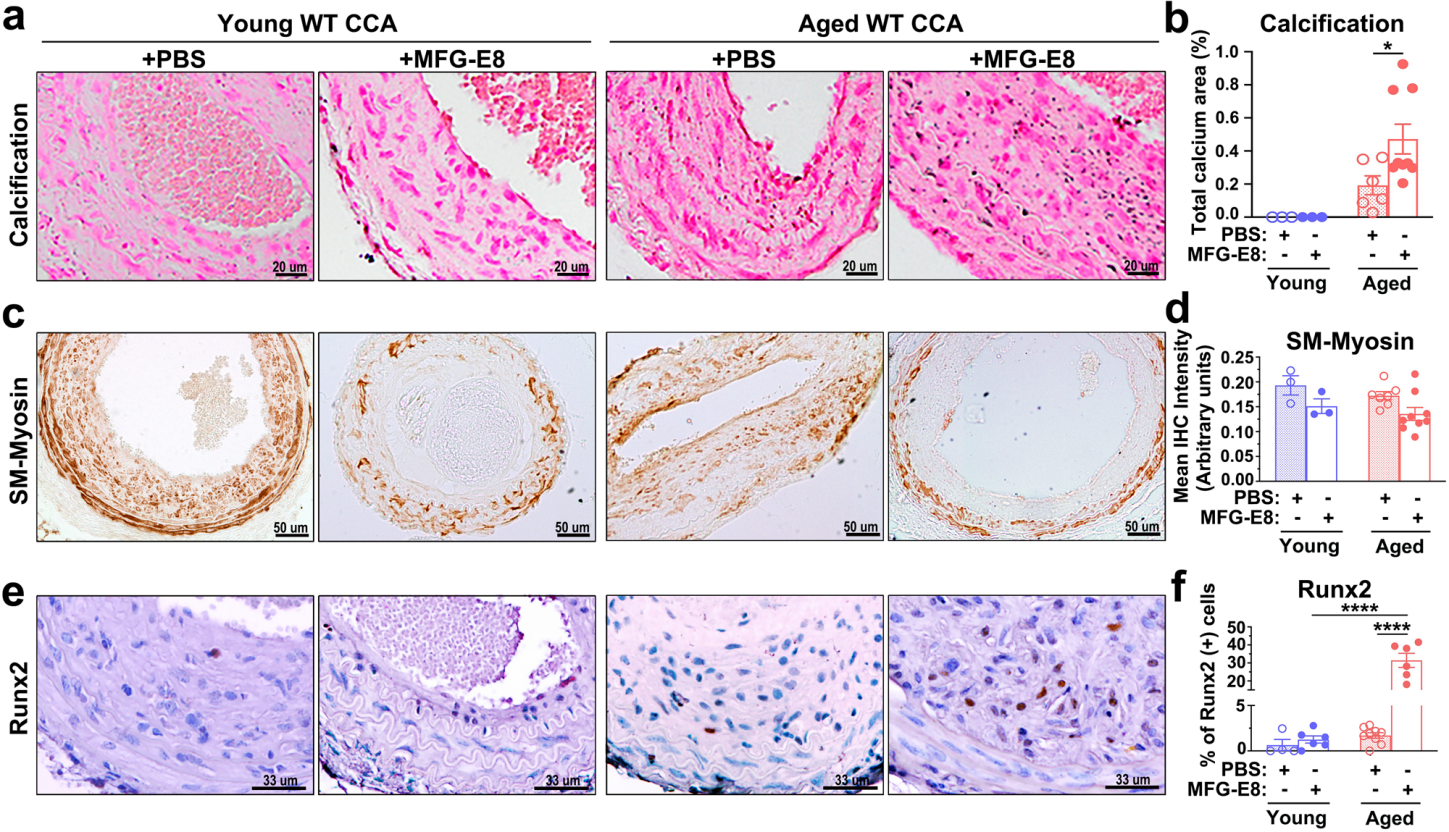
Exogenous application of milk fat globule–epidermal growth factor 8 (MFG-E8) promotes the vascular calcification and osteogenic transdifferentiation of vascular smooth muscle cells in aged mice. Common carotid arteries (CCAs) of young and aged wild-type (WT) mice were ligated to induce vascular remodeling. Pluronic gel containing phosphate-buffered saline (PBS) or rMFG-E8 was applied on CCAs immediately after the ligation. **a**, **b** Paraffin sections of ligated CCAs 21 days postligation were subjected to von Kossa staining. **a** Representative images of CCA sections from four groups of mice indicating calcium deposition in the arterial walls. **b** Quantification of calcified area in the arterial walls (Young + PBS: *n*_mice_ = 3, Young+MFG-E8: *n*_mice_ = 3, Aged+PBS: *n*_mice_ = 6, Aged+MFG-E8: *n*_mice_ = 9). Results are presented as mean ± standard error of the mean (SEM). Each data point is derived from an assessment of three sections of one individual animal. *P < 0.05, as obtained using one-way analysis of variance (ANOVA) followed by Tukey’s multiple comparisons test. **c** Representative immunohistochemistry (IHC) images of smooth muscle myosin (SM-Myosin) in the CCAs of mice 21 days after ligation. Scale bar: 50 μm. **d** Quantitative analysis of the immunostaining intensities of SM-Myosin in the intimal medial area was performed (Young + PBS: *n*_mice_ = 3, Young+MFG-E8: *n*_mice_ = 3, Aged+PBS: *n*_mice_ = 7, and Aged+MFG-E8: *n*_mice_ = 9). Data are presented as mean ± SEM. Each data point was derived from an assessment of three sections from one individual animal. **e** Representative IHC photomicrographs of runt-related transcription factor 2 (Runx2) in the ligated CCAs 21 days after surgery. Scale bar: 33 μm. **f** Quantitative IHC analysis of the percentage of Runx2-positive cells over the total cell number in the intimal medial area (Young + PBS: *n*_mice_ = 4, Young+MFG-E8: *n*_mice_ = 6, Aged+PBS: *n*_mice_ = 9, and Aged+MFG-E8: *n*_mice_ = 6). Data are presented as mean ± SEM. Each data point was derived from an assessment of three sections from one individual animal. ****P < 0.0001, obtained using one-way ANOVA followed by Tukey’s multiple comparisons test.

Our previous study revealed that MFG-E8 expression is elevated in aged CCAs^22^, and vascular calcification is prevalent in aging arteries. Therefore, we subsequently assessed the extent of calcium deposition in the ligated CCAs of young and aged mice. Von Kossa staining indicated some punctate calcium deposits in the ligated arteries of aged mice but not in young mice 21 days after the ligation (Fig. 3a, b). To further investigate the role of MFG-E8 in vascular calcification, exogenous recombinant MFG-E8 (rMFG-E8) was periadventitially delivered onto the ligated CCAs of young and aged mice by using pluronic gel. With the administration of rMFG-E8, the neointima and media of aged CCAs exhibited significant calcium formation compared with those of young arteries (Fig. 3a, b). No sex-dependent difference in the extent of calcium deposition was noted in the aged ligated CCAs with or without treatment with MFG-E8 (Supplementary Fig. 1g). These results further confirm the involvement of MFG-E8 in the pathogenesis of vascular calcification.

### MFG-E8 deficiency attenuates osteogenic transdifferentiation of VSMCs induced by osteogenic stimuli

**Fig. 4:**
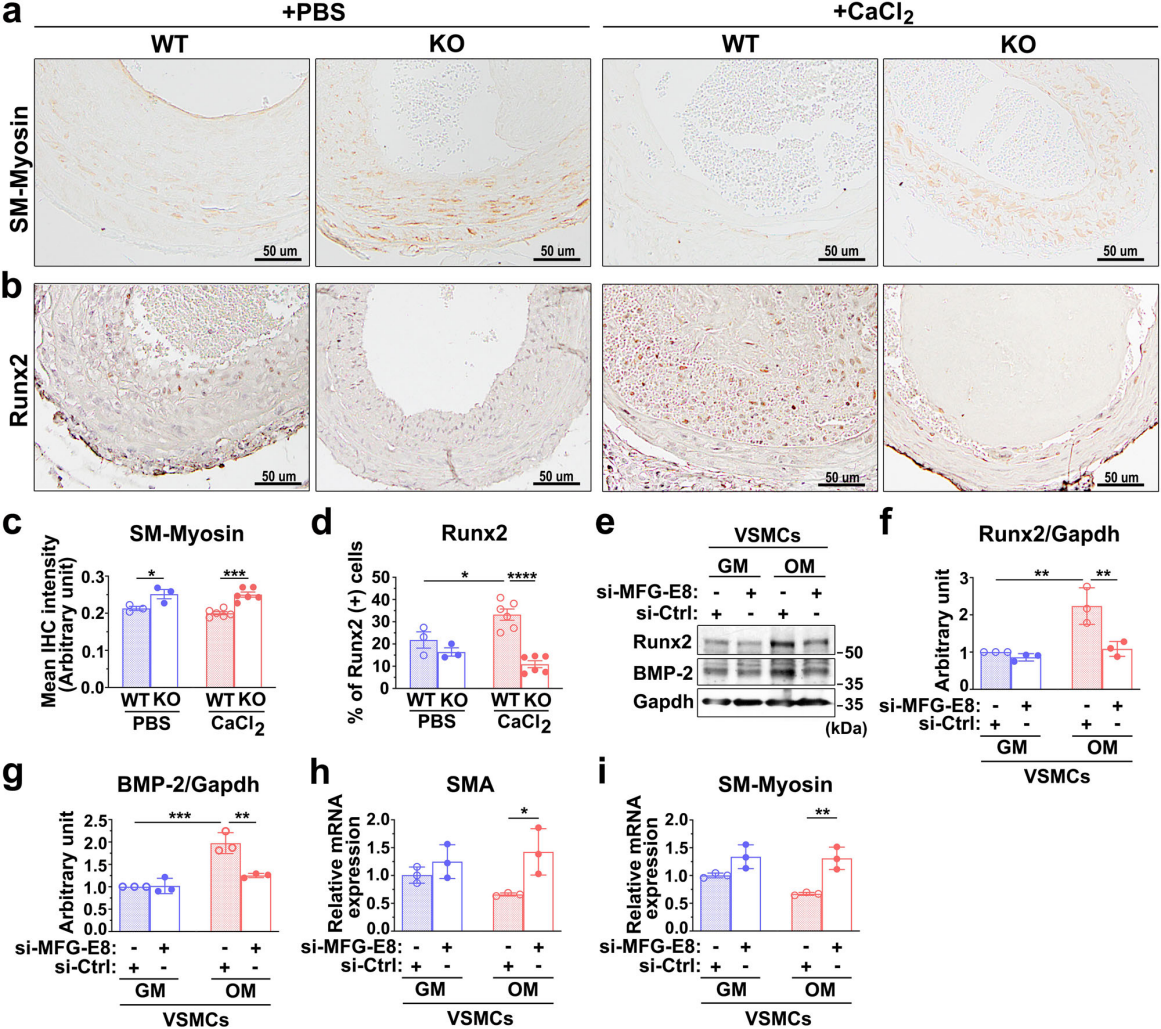
Milk fat globule–epidermal growth factor 8 (MFG-E8) deficiency attenuates osteogenic transdifferentiation of vascular smooth muscle cells (VSMCs) under calcifying conditions. **a**–**d** The common carotid arteries (CCAs) of wild-type (WT) and MFG-E8-knockout (KO) mice were ligated, and phosphate-buffered saline (PBS) and CaCl_2_ (0.4 M) were applied to vessels using Pluronic gel. **a** Representative immunohistochemistry (IHC) images of smooth muscle myosin (SM-Myosin) in the CCAs of mice 21 days after ligation. Scale bar: 50 μm. **b** Representative IHC photomicrographs of runt-related transcription factor 2 (Runx2) in the ligated CCAs 21 days after the surgery. Scale bar: 50 μm. **c** Quantitative analysis of the immunostaining intensities of SM-Myosin in the intimal medial area was performed (WT + PBS: *n*_mice_ = 3, KO + PBS: *n*_mice_ = 3, WT + CaCl_2_: *n*_mice_ = 6, and KO + CaCl_2_: *n*_mice_ = 6). Data are presented as mean ± standard error of the mean (SEM). Each data point is derived from an assessment of three sections of one individual animal. *P < 0.05 and ***P < 0.001, as obtained using one-way analysis of variance (ANOVA) followed by Tukey’s multiple comparisons test. **d** Quantitative IHC analysis of the percentage of Runx2-positive cells over total cell number in the intimal medial area (WT + PBS: *n*_mice_ = 3, KO + PBS: *n*_mice_ = 3, WT + CaCl_2_: *n*_mice_ = 6, KO + CaCl_2_: *n*_mice_ = 6). Data are presented as mean ± SEM. Each data point is derived from an assessment of three sections of one individual animal. *P < 0.05 and ****P < 0.0001, as obtained using one-way ANOVA followed by Tukey’s multiple comparisons test. **e**–**i** A10 VSMCs that underwent 48-h transfection with siRNA against MFG-E8 (si-MFG-E8) or control siRNA (si-Ctrl) were cultured in the growth medium (GM) and osteogenic medium (OM) for 7 days. **e** Immunoblotting of the protein expression of Runx2 and bone morphogenetic protein-2 (BMP-2) in A10 cells was performed. Quantitative analyses of Runx2 (**f**) and BMP-2 (**g**) normalized to glyceraldehyde 3-phosphate dehydrogenase (GAPDH) were conducted (*n* = 3). Data are presented as mean ± standard deviation (SD). Three independent experiments were performed. Each data point is derived from each of the three repeated experiments. **P < 0.01 and ***P < 0.001, as obtained using one-way ANOVA followed by Tukey’s multiple comparisons test. The transcript expression of smooth muscle α-actin (SMA) (**h**) and SM-Myosin (**i**), respectively, was evaluated through quantitative real-time polymerase chain reaction (*n* = 3). Three independent experiments were performed. Data are presented as mean ± SD. Each data point is derived from each of the three repeated experiments. *P < 0.05 and **P < 0.01, as obtained using one-way ANOVA followed by Tukey’s multiple comparisons test.

To clarify why the genetic deletion of MFG-E8 in CCAs and VSMCs prevents calcium deposition in response to osteogenic stimuli, we investigated the underlying molecular mechanisms. Transdifferentiation of VSMCs to osteoblast-like cells is the critical event in the pathogenesis of vascular calcification; therefore, we subsequently evaluated the phenotypes of VSMCs in the CCAs under calcification by conducting an immunohistochemistry (IHC) analysis of SM-Myosin and Runx2. CaCl_2_ application did not alter the expression of SM-Myosin in the injured arterial walls of the WT and KO mice. However, the genetic deletion of MFG-E8 significantly upregulated the expression of SM-Myosin in the ligated CCAs regardless of whether CaCl_2_ was exogenously applied to the vessels (Fig. 4a, c). As expected, an increased number of Runx2-positive cells was observed in the vascular walls of ligated CCAs of the WT mice treated with CaCl_2_ (Fig. 4b, d). By contrast, CaCl_2_ administration to the ligated CCAs of the KO mice did not induce a dramatic alteration in Runx2 expression in the arterial walls compared with the PBS-treated controls (Fig. 4b, d). No sex-dependent differences in the expression of SM-Myosin and the number of Runx2-positive cells were noted in the CaCl_2_-treated arteries of the WT and KO mice (Supplementary Fig. 1c, d). Subsequently, by silencing the expression of MFG-E8 in A10 cells (Supplementary Fig. 3), we evaluated the in vitro necessity of MFG-E8 in the osteogenic transdifferentiation of VSMCs induced by the calcifying medium. In response to the OM, A10 VSMCs transfected with control small interfering RNAs (siRNA) remarkably increased the protein expression of the osteogenic transcription factor, Runx2, and a potent calcification promoting factor, bone morphogenetic protein-2 (BMP-2), as evaluated through immunoblotting (Fig. 4e–g). In addition, the VSMCs cultured in OM tended to attenuate the transcription levels of smooth muscle cell differentiation genes, such as SMA and SM-Myosin (Fig. 4h, i). By contrast, A10 cells treated with siRNA against MFG-E8 exhibited markedly reduced protein levels of Runx2 and BMP-2 (Fig. 4e–g) and the stable transcriptional expression of SMA and SM-Myosin (Fig. 4h, i) during culturing in OM. We also analyzed whether the osteogenic transdifferentiation of VSMCs occurred in the MFG-E8-treated CCAs of aged mice. As depicted in Fig. 3c, d, no statistical difference in the expression of SM-Myosin was observed in the CCAs of young and aged mice with or without the application of rMFG-E8. However, the exogenous administration of MFG-E8 significantly increased the number of Runx2-positive cells in the intima media of the aged arterial walls (Fig. 3e, f). No sex-dependent differences in the expression of SM-Myosin and the number of Runx2-positive cells were noted in the aged CCAs with or without MFG-E8 treatment (Supplementary Fig. 1h, i). These results jointly demonstrate that MFG-E8 drives the osteogenic transdifferentiation of VSMCs in vivo and in vitro in response to osteogenic induction.

### MFG-E8 deficiency reduces the activation of TGF-β1 signaling induced by osteogenic stimuli in VSMCs

**Fig. 5:**
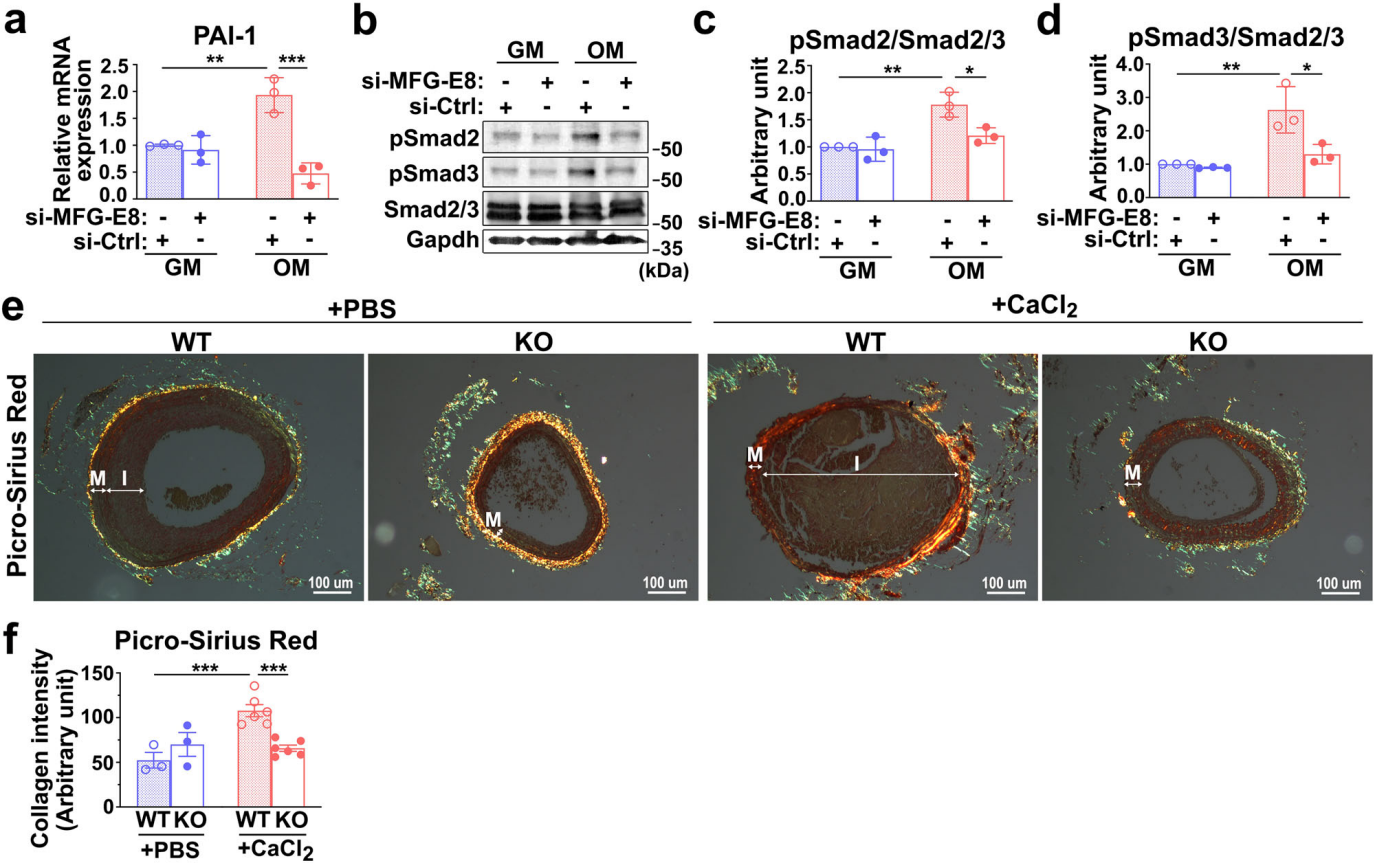
Milk fat globule–epidermal growth factor 8 (MFG-E8) deficiency ameliorates calcifying medium-induced transforming growth factor-β1 (TGF-β1) activation in vascular smooth muscle cells (VSMCs). **a**–**d** A10 VSMCs treated with si-MFG-E8 or control siRNA (si-Ctrl) were cultured in growth medium (GM) and osteogenic medium (OM) for 7 days. **a** The transcript expression of TGF-β1-induced gene PAI-1 was evaluated through quantitative real-time polymerase chain reaction (*n* = 3). Three independent experiments were performed. Data are presented as mean ± standard deviation (SD). Each data point is derived from each of the three repeated experiments. **P < 0.01 and ***P < 0.001, obtained using one-way analysis of variance (ANOVA) followed by Tukey’s multiple comparison test. **b** Western blot analysis of the phosphorylated Smad2 (P-Smad2) and Smad3 (P-Smad3) in VSMCs cultured in GM and OM. Quantitative analyses of P-Smad2 (**c**) and P-Smad3 (**d**) levels normalized to those of total Smad2/3 were conducted, respectively (*n* = 3). Immunoblotting of glyceraldehyde 3-phosphate dehydrogenase was performed to ensure that an equal amount of total proteins was loaded into each sample. Data are presented as mean ± SD. Three independent experiments were performed. Each data point is derived from each of the three repeated experiments. *P < 0.05 and **P < 0.01, as obtained using one-way ANOVA followed by Tukey’s multiple comparisons test. **e**, **f** Common carotid arteries (CCAs) of wild-type (WT) and MFG-E8 knockout (KO) mice were ligated, and phosphate-buffered (PBS) or CaCl_2_ (0.4 M) was applied to the vessels by using pluronic gel. **e** Picrosirius red stain of CCA sections from four experimental groups. Scale bar, 100 μm. The neointima (I) and media (M) of the vessels are indicated. **f** Quantification of the collagen contents by evaluating the intensity as indicated by yellow, orange, and green in the intimal medial area (WT + PBS: *n*_mice_ = 3, KO + PBS: *n*_mice_ = 3, WT + CaCl_2_: *n*_mice_ = 6, KO + CaCl_2_: *n*_mice_ = 6). Data are presented as mean ± standard error of the mean. Each data point is derived from an assessment of three sections of an individual animal. ***P < 0.001, as obtained using one-way ANOVA followed by Tukey’s multiple comparisons test.

The TGF-β1-mediated signaling cascade is widely regarded as a key modulator of vascular calcification^35^. The data suggest that the biomineralization and osteogenic transdifferentiation of VSMCs is regulated by MFG-E8; thus, we examined the involvement of TGF-β1-signaling in MFG-E8-mediated VSMC calcification. To validate the activation of TGF-β1-mediated signaling induced by OM in VSMCs, we evaluated the transcription expression of PAI-1—the downstream gene of TGF-β1 (Fig. 5a)—and the phosphorylation of Smad2/3 (Fig. 5b–d) through quantitative real-time polymerase chain reaction (PCR) and immunoblotting, respectively. The silencing of MFG-E8 expression with siRNA markedly reduced, relative to those of control siRNA, the mRNA expression of PAI-1, and the phosphorylation of Smad2/3 that was induced by calcifying medium (Fig. 5a–d). Additionally, we examined the expression of collagen, which is also the downstream molecule of TGF-β1 and is highly expressed by calcifying cells^36^, through picrosirius red stain. Periadventitial treatment of CaCl_2_ dramatically upregulated the collagen content in the media of WT CCAs but not in that of KO CCAs (Fig. 5e, f). No sex-dependent differences in the collagen levels in the CaCl_2_-treated arteries of the WT and KO mice were noted (Supplementary Fig. 1e). Collectively, these results demonstrate that MFG-E8 promotes TGF-β1 signaling in VSMCs in a procalcifying environment.

### MFG-E8 promotes procalcifying TGF-β1 signaling through its regulation of MMP2 expression

**Fig. 6:**
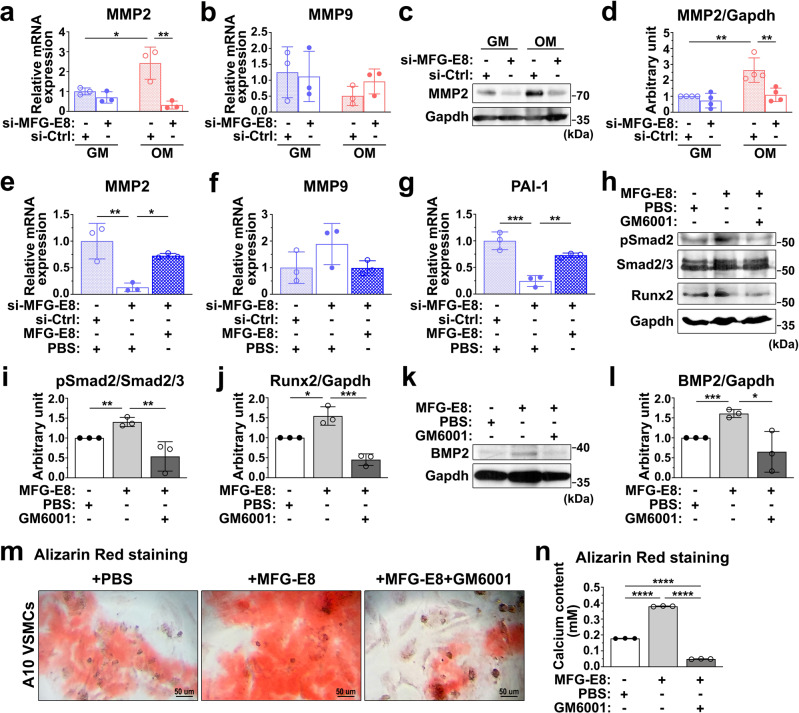
Milk fat globule–epidermal growth factor 8 (MFG-E8) promotes vascular smooth muscle cell (VSMC) calcification and transforming growth factor-β1 (TGF-β1) signaling through upregulating matrix metalloproteinase 2 (MMP2) activity. **a**, **b** A10 VSMCs treated with si-MFG-E8 or control siRNA (si-Ctrl) were cultured in growth medium (GM) and osteogenic medium (OM) for 6 h. The mRNA expression of MMP2 (**a**) and MMP9 (**b**) in VSMCs was evaluated through quantitative real-time polymerase chain reaction (*n* = 3). Three independent experiments were performed. Data are presented as mean ± standard deviation (SD). Each data point was derived from each of the three repeated experiments. *P < 0.05 and **P < 0.01, obtained using one-way analysis of variance (ANOVA) followed by Tukey’s multiple comparison test. **c**, **d** A10 cells transfected with si-MFG-E8 or si-Ctrl were cultured in GM and OM for 7 days. **c** Western blot analysis of MMP2 in VSMCs. **d** Quantitative analysis of MMP2 levels normalized to those of glyceraldehyde 3-phosphate dehydrogenase (GAPDH) was performed (*n* = 3). Data are presented as mean ± SD. Three independent experiments were performed. Each data point was derived from each of the three repeated experiments. **P < 0.01, obtained using one-way ANOVA followed by Tukey’s multiple comparison test. **e**–**g** A10 VSMCs incubated with si-Ctrl, si-MFG-E8, and si-MFG-E8 with recombinant MFG-E8 (rMFG-E8) (100 ng/mL) were cultured in OM for 7 days. The transcript expression levels of MMP2 (**e**), MMP9 (**f**), and PAI-1 (**g**) were evaluated through quantitative real-time polymerase chain reaction (*n* = 3). Data are presented as mean ± SD. Three independent experiments were performed. Each data point was derived from each of the three repeated experiments. *P < 0.05, **P < 0.01, and ***P < 0.001, obtained using one-way ANOVA followed by Tukey’s multiple comparison test. **h**–**n** A10 VSMCs incubated with phosphate-buffered saline (PBS), rMFG-E8, and rMFG-E8 with GM6001 (25 μM) were cultured in OM for 7 days. Immunoblotting of phosphorylated Smad2 (P-Smad2), Smad2/3, runt-related transcription factor 2 (Runx2) (**h**), and bone morphogenetic protein-2 (BMP-2) (**k**) in A10 cells was performed. The quantitative analyses of the levels of P-Smad2 to total Smad2/3 (**i**) and Runx2 and BMP-2 normalized to those of GAPDH (**j**, **l**) were conducted, respectively (*n* = 3). Data are presented as mean ± SD. Three independent experiments were performed. Each data point was derived from each of the three repeated experiments. *P < 0.05, **P < 0.01, and ***P < 0.001, obtained using one-way ANOVA followed by Tukey’s multiple comparison test. **m** Representative microscopic images of the alizarin red stain (ARS) of A10 cells cultured in OM for 7 days. Scale bar: 50 μm. **n** Quantitative analysis of ARS levels in VSMCs (*n* = 3). Three independent experiments were performed, and each experiment was repeated with similar results. The data were derived from one of the representative experiments. ****P < 0.0001, obtained using one-way ANOVA followed by Tukey’s multiple comparison test.

MMP2 is a potent activator of TGF-β1, and studies have revealed that MFG-E8 increases the secretion of MMP2 in macrophages and cancer cells^26,27^. Therefore, we then assessed whether MFG-E8 promoted TGF-β1 activation through the upregulation of MMP-2 expression and activity. We first examined whether MFG-E8 regulated the expression of MMP2 in procalcifying VSMCs. OM significantly induced the mRNA and protein expression of MMP2 (Fig. 6a, c, d) but not MMP9 (Fig. 6b) in the A10 VSMCs treated with the control siRNA. The transfection of A10 cells with siRNA against MFG-E8 remarkably attenuated OM-induced MMP2 expression at the mRNA and protein levels. To clarify the regulation of MMP2 in MFG-E8-mediated TGF-β1 activation, we first evaluated the effect of rMFG-E8 on TGF-β1 activation in VSMCs. The treatment of A10 cells cultured in OM with rMFG-E8 (100 ng/mL) rescued the attenuation of mRNA levels of MMP2 and PAI-1 induced by siRNA against MFG-E8 (Fig. 6e, g), but not that of MMP9 (Fig. 6f). The exogenous application of rMFG-E8 to A10 VSMCs significantly enhanced the Smad2 phosphorylation (Fig. 6h, i), Runx2 (Fig. 6h, j), and BMP-2 expression (Fig. 6k, l), and calcium deposition (Fig. 6m, n) induced by OM. These results indicated that exogenous MFG-E8 was capable of upregulating MMP2 expression, activating TGF-β1-mediated signaling, and potentiating osteogenic transdifferentiation and biomineralization in VSMCs. The treatment of A10 cells cultured in OM with GM6001, an MMP inhibitor, attenuated rMFG-E8-enhanced Smad2 phosphorylation (Fig. 6h, i), Runx2 (Fig. 6h, j), and BMP-2 protein expression (Fig. 6k, l), and VSMC calcification (Fig. 6m, n), demonstrating that MFG-E8 induced TGF-β1-mediated signaling, osteogenic transdifferentiation, and VSMC biomineralization through its regulation of MMP2 activity.

Elastin fragmentation, degradation, and the resulting elastin-derived peptides are assumed to be the key events that initiate vascular calcification^37^. In addition to activating TGF-β1-mediated signaling, evidence suggests that MMPs contribute to vascular calcification through the degradation of elastic fibers^37^. To determine whether MFG-E8 promotes VSMC biomineralization and osteogenic transdifferentiation also through MMP2-mediated elastolysis, elastin fragmentation was quantitated in the injured CCAs. Verhoeff–van Gieson (VVG) staining of ligated CCAs conducted 3 weeks after CaCl_2_ treatment revealed that the presence of only ligation injury induced no obvious elastin fragmentation in the carotid arteries of WT and MFG-E8-KO mice, whereas the CaCl_2_-treated CCAs of WT and KO littermates exhibited obvious elastin fragmentation; however, no significant difference was noted between WT and KO CCAs after CaCl_2_ treatment (Supplementary Fig. 4a, b). We then examined the contents of elastin in calcifying vessels through IHC. The periadventitial application of CaCl_2_ markedly decreased the level of elastin in the arterial walls of the WT and MFG-E8-KO mice; however, no difference was observed between the WT and KO CCAs (Supplementary Fig. 4c, d). We also evaluated the integrity of elastin fibers in the ligated CCAs of aged mice. As depicted in Supplementary Fig. 5a, b, no significant elastin fragmentation was observed in the ligated CCAs of aged WT mice with or without MFG-E8 treatment. Consistently, the exogenous administration of rMFG-E8 into the ligated arteries of aged mice did not alter the expression of elastin in the arterial wall compared with the vessels treated with PBS (Supplementary Fig. 5c, d). Collectively, these results demonstrate that MFG-E8-mediated MMP2 expression is not involved in elastin-associated vascular calcification.

### MFG-E8 regulates the osteogenic transdifferentiation of VSMCs and TGF-β1 activation through β1 integrin ligation

**Fig. 7:**
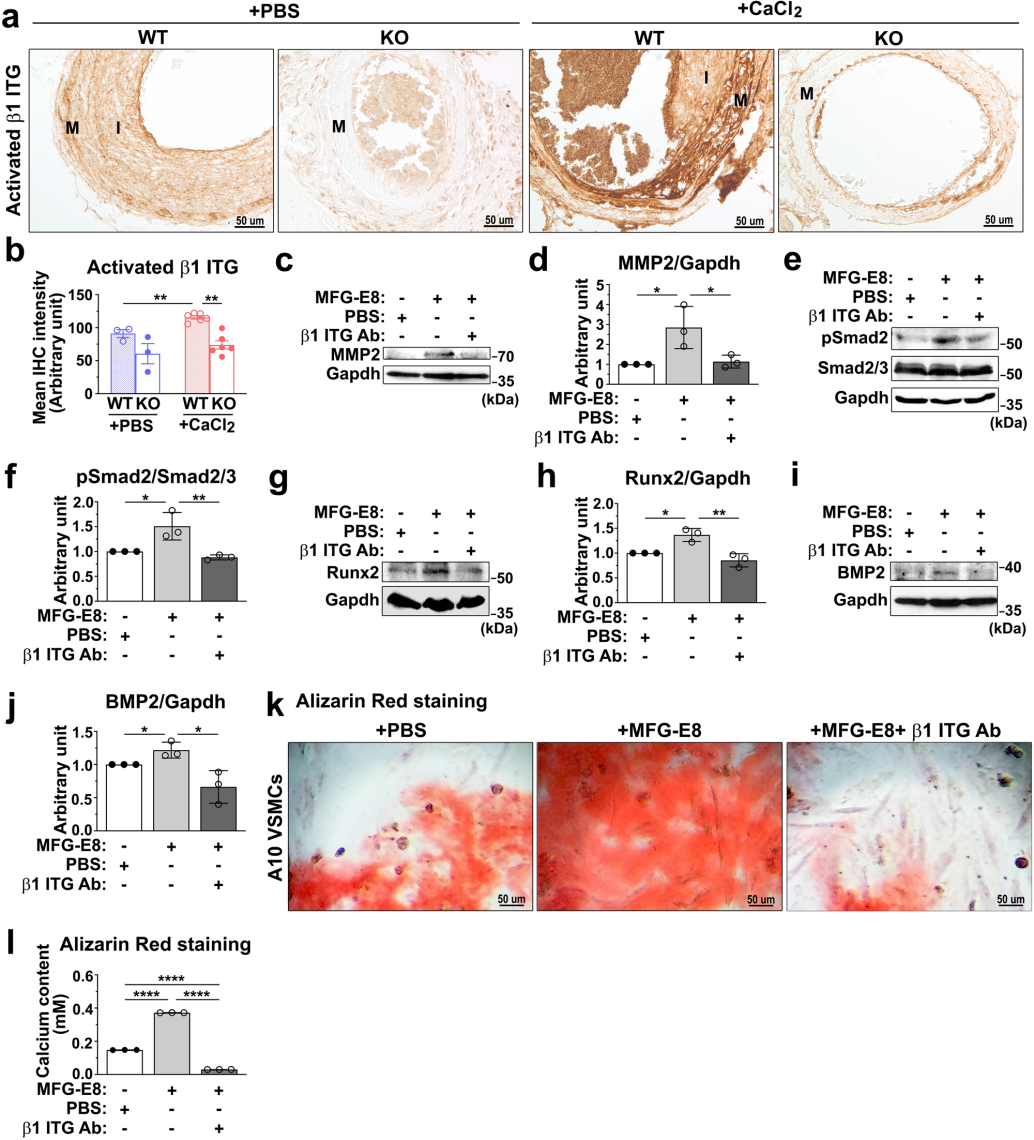
Milk fat globule–epidermal growth factor 8 (MFG-E8) promotes the osteogenic transdifferentiation of vascular smooth muscle cells (VSMCs) and activation of the transforming growth factor-β1 (TGF-β1)-mediated signaling cascade through β1 integrin engagement. **a**, **b** Ligated common carotid arteries (CCAs) of wild-type (WT) and MFG-E8-knockout (KO) mice were treated with phosphate-buffered saline (PBS) and CaCl_2_ (0.4 M) by using pluronic gel. **a** Representative immunohistochemistry (IHC) microphotographs of activated β1 integrin in the CCAs of mice 21 days after ligation. Scale bar: 50 μm. **b** Quantitative analysis of the IHC intensities of activated β1 integrin in the intimal medial area (WT + PBS: *n*_mice_ = 3, KO + PBS: *n*_mice_ = 3, WT + CaCl_2_: *n*_mice_ = 6, KO + CaCl_2_: *n*_mice_ = 6). Data are presented as mean ± standard error of the mean. Each data point is derived from an assessment of three sections of an individual animal. **P < 0.01, as obtained using one-way analysis of variance (ANOVA) followed by Tukey’s multiple comparisons test. **c**–**l** A10 cells treated with PBS, rMFG-E8 (100 ng/mL), and rMFG-E8 with β1 inhibitory antibody (50 μg/mL) were cultured in osteogenic medium (OM) for 7 days. Immunoblotting was performed to assess the protein expression of matrix metalloproteinase 2 (MMP2) (**c**), phosphorylated Smad2 (P-Smad2) (**e**), runt-related transcription factor 2 (Runx2) (**g**), and bone morphogenetic protein-2 (BMP-2) (**i**) in A10 cells. The quantitative analyses of the levels of MMP2, Runx2, and BMP-2 normalized to those of glyceraldehyde 3-phosphate dehydrogenase (GAPDH) (**d**, **h**, **j**, respectively), and P-Smad2 to total Smad2/3 (**f**) were conducted (*n* = 3). Data are presented as mean ± standard deviation (SD). Three independent experiments were performed. Each data point was derived from each of the three repeated experiments. *P < 0.05 and **P < 0.01, obtained using one-way ANOVA followed by Tukey’s multiple comparison test. **k** Representative microscopic images of the alizarin red stain (ARS) of 7-day cultured A10 cells. Scale bar: 50 μm. **l** Quantitative analysis of ARS levels in A10 cells (*n* = 3). Data are presented as mean ± SD. Three independent experiments were performed, and each experiment was repeated with similar results. The data were derived from one of the representative experiments. ****P < 0.0001, obtained using one-way ANOVA followed by Tukey’s multiple comparison test.

MFG-E8 has been reported to mediate a variety of cell behaviors through integrin signaling. Evidence has indicated that genetic deletion or functional impairment of β1 integrin in osteoblasts led to defective bone formation and reduced osteoblast differentiation and mineralization^38,39^. In a recent study, α8β1 integrin was highlighted as a novel receptor for MFG-E8 in smooth muscle cells^40^, suggesting that MFG-E8-mediated osteogenic transdifferentiation of VSMCs and TGF-β1 activation may be attributable to β1 integrin engagement and activation. We first assessed the activation of β1 integrin in the calcifying CCAs of WT and KO mice by immunostaining the vessels with the antibodies specific to activated β1 integrin. Compared with the ligated CCAs of KO mice, those of WT mice exhibited higher IHC intensity of activated β1 integrin (Fig. 7a, b). As expected, CaCl_2_ treatment remarkably increased the levels of activated β1 integrin in WT CCAs but not in KO CCAs (Fig. 7a, b). No sex differences in the levels of activated β1 integrin were observed in the CaCl_2_-treated CCAs of the WT and KO mice (Supplementary Fig. 1f). To directly examine the involvement of β1 integrin in MFG-E8-mediated osteogenic transdifferentiation and TGF-β1 activation in VSMCs, we interrupted MFG-E8 and β1 integrin ligation using the β1 integrin inhibitory antibody. The pretreatment of A10 cells cultured in OM with the neutralizing anti-β1 integrin antibody substantially attenuated the increased expression of MMP2 induced by exogenous MFG-E8 (Fig. 7c, d). Furthermore, the functional blockade of β1 integrin with the blocking antibody significantly reduced MFG-E8-elicited Smad2 phosphorylation (Fig. 7e, f), Runx2 (Fig. 7g, h), and BMP-2 expression (Fig. 7i, j)—as evidenced through immunoblotting—and VSMC biomineralization (Fig. 7k, l). Together, these findings indicated that β1 integrin engagement is necessary for MFG-E8-induced MMP2 expression, which activates TGF-β1-mediated signaling and the subsequent osteogenic transdifferentiation and calcification of VSMCs.

## Discussion

The stiffening of the conduit arteries is one of the characteristics of arterial aging^41^. The stiffness of the artery is largely dependent on the elevated collagen to elastin ratio and calcium deposition within the vessel wall^42,43^. MFG-E8 is highly expressed in aging arteries and is thus a useful biomarker for arterial aging^20,21^. In one study, a positive correlation was observed between the serum level of MFG-E8 and arterial stiffness in older adult patients with type 2 diabetes mellitus or CKD^23,24^, suggesting a link between MFG-E8 and vascular stiffness. However, research on the mechanisms through which MFG-E8 promotes arterial stiffness is lacking. In this study, we provided direct in vivo and in vitro evidence demonstrating that MFG-E8 promotes VSMC biomineralization in response to procalcifying stimuli, leading to the accumulation of calcium in the arterial walls, a key contributor to the loss of arterial compliance^44,45^.

Integrins, a large family comprising transmembrane receptors for ECM and other Arg-Gly-Asp (RGD)–containing proteins (such as MFG-E8), are heterodimers composed of one α and one β subunit^46^. The ligation of integrin by appropriate ligand proteins causes conformational changes in integrin, which in turn initiate integrin activation and elicit various downstream signaling pathways to regulate cell behaviors^47^. Integrins are involved in both physiological and pathological osteogenic differentiation. Ligation of β1 integrin in osteoblasts promotes osteoblast differentiation and bone formation^38,39,48^, whereas ligation of αvβ3 integrin aids osteoblast migration but impedes osteoblast differentiation^49^, indicating that engagement of distinct integrins initiates particular cell responses in the same cells. MFG-E8 is a ligand for αvβ3, αvβ5, and α8β1 integrins^40,50,51^. Exogenous rMFG-E8 promotes VSMC migration through αvβ5 integrin signaling^21^, and MFG-E8-α8β1 integrin interaction regulates SMC contraction^51^. In the present study, we demonstrate that the functional blockade of β1 integrin successfully attenuates MFG-E8-induced MMP2 expression and the subsequent activation of TGF-β1. Our findings are the first to identify the roles of the MFG-E8–β1 integrin axis in VSMC biomineralization and osteogenic transdifferentiation.

In this study, we determined that MFG-E8 promotes VSMC biomineralization and vascular calcification by regulating TGF-β1 signaling. TGF-βs are produced by SMC-like cells^52^ and have three different isoforms, namely TGF-β1, TGF-β2, and TGF-β3, of which TGF-β1 is the most critical for the cardiovascular system^53^. Active TGF-β1 can transduce signals through two distinct type I receptors, activin-like kinase 1 (ALK1) and ALK-5^13,14^. ALK1 and ALK5 induce the phosphorylation of Smad1/5 and Smad2/3, respectively^13^. A study revealed that TGF-β1 induces vascular calcification through the ALK5/Smad2/3 signaling axis^54^. Phosphorylated Smad2/3 complexes with Smad4 cotranslocate into the nucleus, promoting the osteogenic transdifferentiation of VSMCs and calcification through induction of the transcriptional activation of a number of osteogenic genes^52,54,55^. Additionally, our results demonstrated that MFG-E8 promotes TGF-β1-mediated collagen expression. Furthermore, ECM proteins, such as fibronectin and collagen I, are known to promote VSMC calcification^36^. Together, MFG-E8 may promote VSMC biomineralization and vascular calcification through the TGF-β1-regulated phenotypic switch of VSMC into osteoblast-like cells and ECM protein upregulation.

The use of animal models in vascular calcification intervention has deepened our understanding of the molecular mechanisms of the disease in the broad physiological context, which is crucial for developing therapeutic options for patients with vascular calcification. Several rodent models have been verified to mimic the pathological processes of vascular calcification in humans. For CKD induction, one of the etiologies of vascular calcification, surgical intervention to produce renal insufficiency, such as 5/6 nephrectomy and ureteral obstruction, is performed^56–58^. The major limitations of using surgery-induced CKD models have been a considerable effort required, surgery-dependent variation as well as the postoperative complications^58,59^. Dietary intake of a nephrotoxic adenine diet generates severe CKD, with no surgical procedures required^58,59^. However, severe weight loss and high biological variability in calcification progression have been noted^58,59^. In some genetically modified mouse models with impaired calcium–phosphate and lipid metabolism, the mineralization of the vascular wall can develop spontaneously or be reinforced through the feeding of a special dietary supplement^58–60^. The disadvantage is that vascular calcification is only prominent in aged animals, and a long study duration is thus required. In this study, we established a mouse model of local arterial calcification through the periadventitial introduction of CaCl_2_ to CCAs. This short-term, localized insult induced accelerated and extensive arterial calcification in the mouse CCAs that resembled the medial calcification occurring in aging arteries and many cardiovascular diseases. This model is advantageous for its speed, ease of operation, and repeatability. However, local arterial calcification may not comprehensively reflect the pathological condition in humans, because calcification is a systemic disease affecting the whole body. Additionally, we applied ligated CCAs in our modified mouse models. Carotid calcification has been recognized as an indication of aging^61^. In addition to prompting the phenotypic switch of VSMCs from synthetic to osteogenic, ligation injury-induced neointimal hyperplasia in the arteries, mimicking the increased intima-media thickness in aging arteries.

Accumulating evidence indicates that chronic inflammation, commonly observed in older adults and those with atherosclerosis, diabetes, and CKD^62,63^, is the key contributor to the pathogenesis of cardiovascular calcification. Another study of ours revealed that MFG-E8 is highly expressed in aging arteries, playing a proinflammatory role in arterial aging^22^. In the present study, we verified that MFG-E8 promotes MMP2 expression in a β1 integrin-dependent manner. Monocyte chemoattractant protein-1 (MCP-1), the downstream molecule of MFG-E8^20^ and a member of the chemokine family, is induced by integrin ligation^64^ and plays a pivotal role in inflammatory processes^65^. Moreover, MCP-1 can induce the activation of TGF-β by activating MMP-2^8,26^. These results indicate that MFG-E8 may upregulate MMP2 expression through the β1 integrin/MCP-1 signaling axis in a proinflammation-dependent manner. We infer that the MFG-E8 mechanism for regulating vascular calcification is the exacerbation of inflammatory reactions, leading to subsequent MMP2-mediated TGF-β1 activation.

**Fig. 8:**
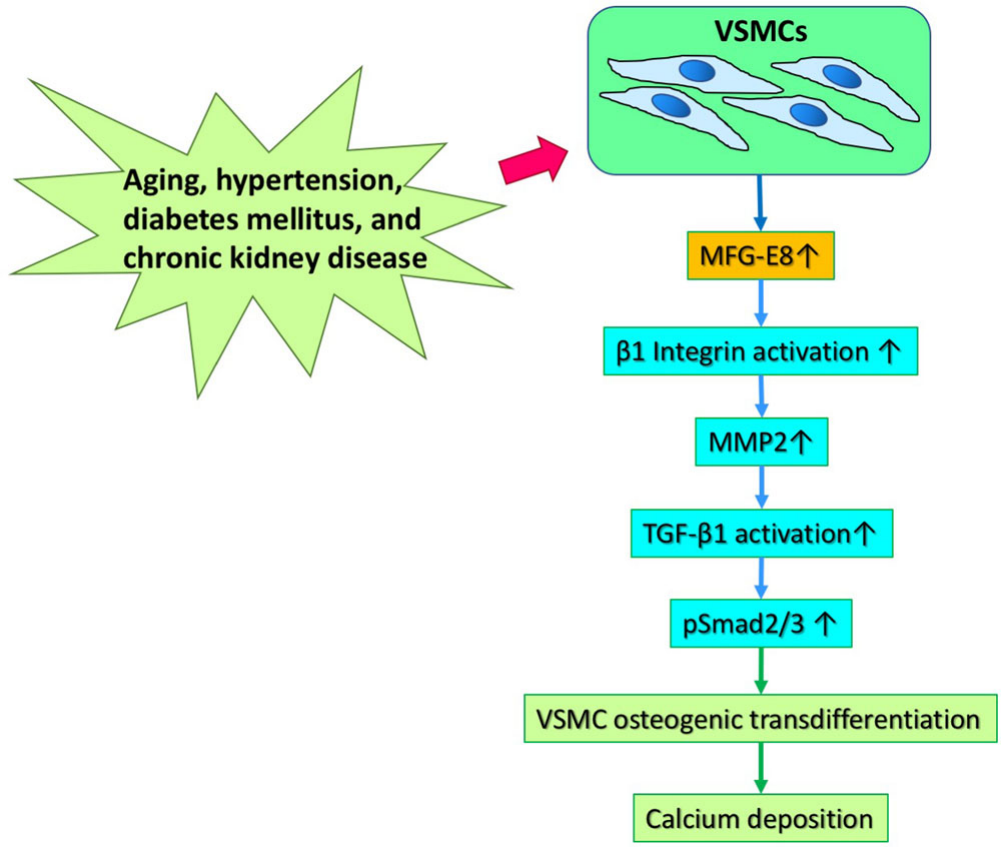
Proposed model in which milk fat globule–epidermal growth factor 8 (MFG-E8) promotes vascular smooth muscle cell (VSMC) osteogenic transdifferentiation and vascular calcification by activating transforming growth factor-β1 (TGF-β1) signaling. MMP2 matrix metalloproteinase 2.

In conclusion, our study demonstrates that MFG-E8 is a critical positive regulator of vascular calcification. Deletion of MFG-E8 reduces the extent of ectopic calcium deposition in a CaCl_2_-induced arterial calcification model. Our findings indicate that MFG-E8 promotes β1 integrin-dependent MMP2 expression and the subsequent TGF-β1 activation in response to osteogenic stimuli, which leads to VSMC osteogenic transdifferentiation and biomineralization (Fig. 8). These findings suggest that MFG-E8 silencing is a potentially promising therapeutic strategy for the treatment of vascular calcification.

## Methods

### Animal study

Male and female WT and MFG-E8-KO mice (6–8 weeks old; Riken BioResource Center, Saitama, Japan)^66^ backcrossed to the FVB/NJNarl background were subjected to a modified vascular calcification model induced by CaCl_2_. Animals were anesthetized with isoflurane inhalation. The left CCA was exposed, dissected free from the surrounding connective tissue, and completely ligated using a 6-0 silk suture proximal to the carotid bifurcation^67,68^. Subsequently, 50 μl of 20% Pluronic gel containing CaCl_2_ (0.4 M) was introduced through periadventitial administration to the carotid artery immediately after complete carotid artery ligation^22,67^. In some experiments, 18-month-old male and female FVB/NJNarl WT mice were subjected to complete CCA ligation followed by periadventitial application of rMFG-E8 (R&D Systems, Minneapolis, MN, USA) using Pluronic gel. All animal procedures were approved by the Institutional Animal Care and Use Committee of Chang Gung University (Approval no: CGU108-210 and CGU15-098) and were conducted in accordance with relevant institutional guidelines for animal research.

### Cell culture and treatment

A10 VSMCs were acquired from Bioresource Collection and Research Center (Hsinchu, Taiwan), and primary VSMCs were enzymatically isolated from the aortas of WT and MFG-E8-KO mice as described in our previous study^22^. A10 cells and primary VSMCs were grown in GM consisting of Dulbecco’s Modified Eagle Medium (DMEM), which contained glucose (4.5 g/L; Corning, Glendale, AZ, USA) supplemented with 10% fetal bovine serum and penicillin–streptomycin solution (penicillin [100 IU] and streptomycin [100 µg/mL]; Corning) under 5% CO_2_. For MFG-E8 silencing, siRNAs (GE Healthcare Dharmacon, Lafayette, CO, USA) were added to A10 cells. To inhibit the activities of MMPs, A10 VSMCs were exposed to GM6001 (25 μM, Merck Millipore, Burlington, MA, USA) for 24 h prior to the treatment with PBS or rMFG-E8 (100 ng/mL) in GM. For the integrin function-blocking assay, the A10 cells were incubated with 50 μg/mL of β1 integrin inhibitory antibody (Ha2/5; BD Pharmingen, San Jose, CA, USA) for 4 h prior to treatment with rMFG-E8 for the indicated periods.

### In vitro induction of VSMC biomineralization and calcium quantification

For calcification induction, A10 and primary VSMCs (passages 3–5) were seeded on a coverslip and grown in GM until they reached 80% confluence. They were then transferred to OM consisting of DMEM containing CaCl_2_ (2.85 mmol/L), β-glycerophosphate disodium salt hydrate (5 mmol/L), 10% fetal bovine serum, and penicillin–streptomycin for 7 days. Media were replaced every other day. After the medium was removed, the cells were washed using PBS, fixed with 10% formalin, and stained with alizarin red S (ARS) solution (ScienCell Research Laboratories, Carlsbad, CA, USA) for 30 min. After images were captured, calcified minerals were extracted with 10% acetic acid and neutralized using 10% ammonium hydroxide. ARS standards, ranging from 30 µM to 4 mM, were prepared. Absorbance of the sample and standards was measured at 405 nm using a SpectraMax M5 microplate reader (Molecular Devices, Sunnyvale, CA, USA). Based on the calibrated optical density (OD)_405nm_ value of the ARS standard, a standard curve was plotted as a function of ARS concentration. The ARS concentrations in the samples were calculated according to the equation of the trend line.

### Tissue collection and processing

CCAs harvested from mice 21 days after surgery were formalin-fixed and embedded in paraffin and cut into 5-μm-thick sections. Because our previous study demonstrated that the most prominent vascular remodeling occurs within the first millimeter proximal to the ligature^69^, sections located at 200-μm intervals within this segment were selected for the following analyses.

### Von Kossa stain

Calcium deposition in CCAs was investigated using von Kossa staining. Formalin-fixed paraffin sections were incubated with 1% silver nitrate solution under exposure to ultraviolet light for 2 h and counterstained with 0.1% nuclear fast red. For quantitative analysis, digital images of the paraffinized sections were captured, and the positive staining area for calcium particles was obtained using an automated programmed segmentation procedure in Image-Pro Plus software (Media Cybernetics, Rockville, MD, USA). The intimal medial area was traced manually, and its positive area was assessed as a percentage of the total traced area.

### Picrosirius red stain

After the sections were deparaffinized, they were stained using a picrosirius red stain kit (Abcam, Cambridge, UK) according to the manufacturer’s instructions. The sections were observed using an Olympus AX70 light microscope with a polarized filter (Melville, NY, USA). Images were processed using Photoshop CC (Adobe, San Jose, CA, USA).

### IHC and quantitative analysis

For each mouse, three sections located at 200-μm intervals within the first millimeter proximal to the ligature were selected for IHC analysis. Sections were incubated with the antibodies of anti-SM-Myosin (1:1000; Biomedical Technologies, Stoughton, MA, USA), anti-Runx2 (1:1000; Cell Signaling Technology, Danvers, MA, USA), anti-active β1 integrin (1:1000; Merck Millipore, Burlington, MA, USA), and elastin (1:1500; Santa Cruz, Dallas, TX, USA). The sections were then incubated with appropriate biotinylated secondary antibodies, after which analysis using an avidin–biotin immunoperoxidase system (Vector Laboratories, Burlingame, CA, USA) was conducted. A liquid diaminobenzidine substrate chromogen system (Dako, Carpinteria, CA, USA) was used for detection. Quantitative IHC analysis was performed on digital images captured with a ×20 objective, and color digital images were converted to grayscale. The average OD in the intimal medial area in each section was measured using Image-Pro Plus.

### Immunofluorescence analysis and quantification of fluorescence intensity

To determine the expression of MFG-E8 in the mouse CCAs, the paraffinized sections from within the first millimeter proximal to the ligature were incubated with anti-MFG-E8 (1:200; R&D Systems, Minneapolis, MN, USA), followed by an additional incubation with Alexa Fluor 488-conjugated secondary antibodies (1:100; Thermo Fisher Invitrogen, Waltham, MA, USA). Confocal images were captured using a ZEISS LSM 780 confocal scanning microscope (Oberkochen, Germany), and the fluorescence images were converted to grayscale images using Photoshop CC. The mean fluorescence intensities of MFG-E8 were analyzed using Image-Pro Plus.

### VVG elastic stain and quantification of elastin fragmentation

Paraffin sections from within the first millimeter proximal to the ligature were stained with VVG elastic stain. Elastin fragmentation is defined as the presence of free ends in a continuous elastin fiber. The number of elastin breaks in each section was determined.

### Western blotting

The siRNA-transfected and rMFG-E8-treated A10 cells and primary VSMCs derived from the WT and MFG-E8-KO mice were homogenized in a 1× Laemmli sample buffer (Sigma-Aldrich, St. Louis, MO, USA). Cell lysates were electrophoresed on sodium dodecyl sulfate–polyacrylamide gel electrophoresis system under reducing conditions and then transferred onto nitrocellulose membranes. Primary antibodies of MFG-E8, Runx2, phosphorylated Smad2, phosphorylated Smad3, Smad2/3 (Cell Signaling), BMP-2 (GeneTex, Irvine, CA, USA), and glyceraldehyde 3-phosphate dehydrogenase (GAPDH, Sigma-Aldrich) were employed in immunoblot analyses, as previously described^70^. Quantitative analysis was performed using densitometry with ImageJ software (National Institutes of Health, Bethesda, MD, USA).

### Quantitative real-time PCR

Total RNA from cells was isolated using TRIzol (Invitrogen, Waltham, MA, USA), and each sample was reverse transcribed using a First Strand cDNA Synthesis Kit (GE Healthcare Life Sciences, Chicago, IL, USA) according to the manufacturer’s instructions. Quantitative real-time PCR was performed using various sets of quantitative PCR primers (Supplementary Table I) and a SensiFAST SYBR No-ROX Kit (Bioline, London, UK) on a CFX96 real-time PCR detection system (Bio-Rad, Hercules, CA, USA). Each quantitative real-time PCR experiment was performed at least thrice. The representative results were expressed as a fold change relative to the control using the standard 2^–ΔΔCt^ method^71^, where Ct represents the number of cycles required to reach the threshold for the target gene subtracted from the number of cycles required to reach the threshold for a control housekeeping gene (in this case, GAPDH).

### Statistics and reproducibility

After we conducted tests for normality and equal variance, appropriate statistical analyses were performed; relevant descriptions are provided in the legend of each figure. For the animal studies, the number of animals is specified in each figure legend, and the data are presented as the mean ± standard error of the mean. All in vitro experiments were performed at least thrice, and the data are presented as the mean ± standard deviation. For data analysis, Student’s *t* test or one-way analysis of variance, followed by Tukey’s multiple comparison post hoc test was conducted using GraphPad Prism (GraphPad, San Diego, CA, USA). For nonnormally distributed data, the nonparametric Mann–Whitney test or Kruskal–Wallis test, followed by Dunn’s post hoc test, was employed. Results with a *P* value of < 0.05 were considered significant.

### Reporting summary

Further information on research design is available in the Nature Research Reporting Summary linked to this article.

## Supplementary information

Supplementary information

Description of Additional Supplementary Files

Supplementary Data 1

Reporting Summary

## Data Availability

The data that support the findings of this study are available from the corresponding author upon reasonable request. The original, uncropped blot images can be found in Supplementary Figs. 6–10. Source data underlying the graphs are provided in Supplementary Data 1.
