# Supplementary material for: MFG-E8 promotes osteogenic transdifferentiation of smooth muscle cells and vascular calcification by regulating TGF-β1 signaling
Source: Commun Biol. 2022 Apr 19;5:364. doi: 10.1038/s42003-022-03313-z (PMC9018696; doi:10.1038/s42003-022-03313-z)
Supplement: Supplementary file 1 — Supplementary information [file 42003_2022_3313_MOESM1_ESM.pdf]

SUPPLEMENTARY INFORMATION

MFG-E8 promotes osteogenic transdifferentiation of smooth muscle cells and vascular calcification by regulating TGF-β1 signaling

Hou-Yu Chiang, PhD[1,2,3*], Pao-Hsien Chu[3,4,5*], Shao-Chi Chen[1], Ting-Hein Lee, PhD[1,2,3]

[*] These authors contributed equally

Running title: MFG-E8 regulates vascular calcification

Author affiliations

[1]Department of Anatomy, College of Medicine, Chang Gung University, Taoyuan, Taiwan

[2]Graduate Institute of Biomedical Sciences, College of Medicine, Chang Gung University, Taoyuan, Taiwan

[3]Division of Cardiology, Department of Internal Medicine, Chang Gung Memorial Hospital, Linkou, Taiwan

[4]College of Medicine, Chang Gung University, Taoyuan, Taiwan

[5]Institute of Stem Cell and Translational Cancer Research, Chang Gung Memorial Hospital, Linkou, Taiwan

Address correspondence to:

Ting-Hein Lee, Ph.D.

Department of Anatomy

College of Medicine

Chang Gung University

259 Wenhua 1st Rd., Guishan Dist., Taoyuan City 33302, Taiwan

Tel: +886-3-2118800, ext. 3880; Fax: +886-3-2118112

Email: tingheinlee@mail.cgu.edu.tw

Supplementary figures and figure legends

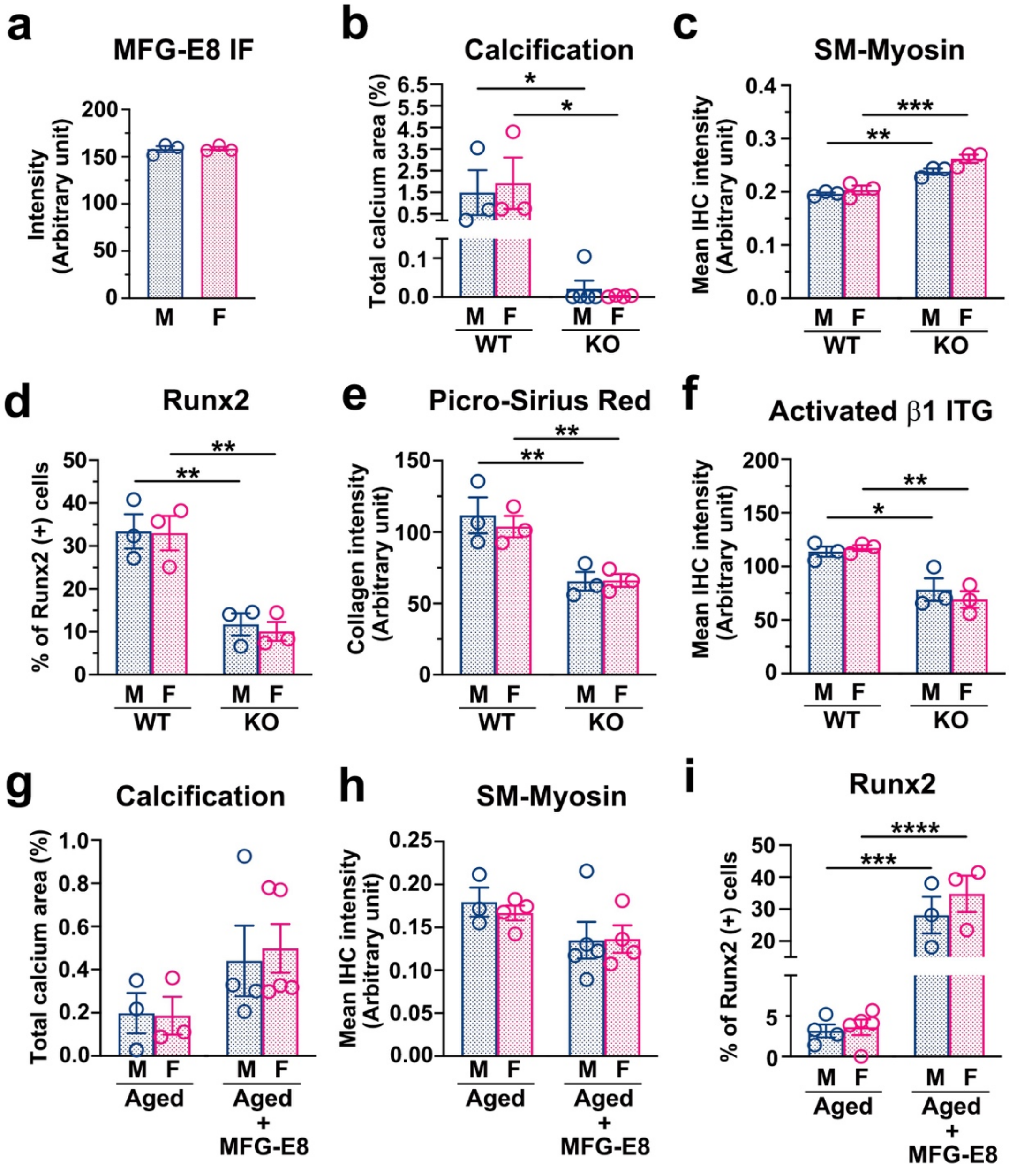

Supplementary Figure 1. MFG-E8 does not elicit sex-dependent differences in vascular calcification. (a)–(f) Ligated common carotid arteries (CCAs) of wild-type (WT) and MFG-E8-knockout (KO) mice were treated with Pluronic gel containing 0.4 M $CaCl_2$ for 21 days to induce vascular calcification. (a) Immunofluorescence analysis of MFG-E8 in the arterial walls of male (M) and female (F) WT mice was performed. Quantification of fluorescence intensities of MFG-E8 in CCAs (WT male: n_{mice} =3, and WT female: n_{mice} =3). Results are

presented as mean ± standard error of the mean (SEM). (b) Von Kossa stain was performed on the CCA sections from male (M) and female (F) WT and KO mice. Quantification of the calcified area in the arterial walls (WT male: n_{mice} =3, WT female: n_{mice} =3, KO male: n_{mice} = 5, and KO female: n_{mice} = 4). Quantitative analysis of the immunostaining intensities of smooth muscle myosin (SM-Myosin) (c) and runt-related transcription factor 2 (Runx2) (d) in the intimal medial area (n_{mice} =3 for each experimental group). (e) Picrosirius red stain of CCA sections was performed to quantify collagen levels through evaluation of the intensity, as indicated in yellow, orange, and green in the intimal medial area (n_{mice} =3 for each experimental group). (f) Quantitative analysis of the immunostaining intensities of activated β1 integrin in the intimal medial area (n_{mice} =3 for each experimental group). (g)–(i) CCAs of aged WT mice were ligated to induce vascular remodeling. Pluronic gel containing rMFG-E8 was applied on some CCAs immediately after ligation. (g) Von Kossa stain was conducted on CCA sections from aged male (M) and female (F) mice with or without MFG-E8 treatment. Quantification of calcified area in the arterial walls (Aged male: n_{mice} = 3, Aged female: n_{mice} = 3, Aged+MFG-E8 male: n_{mice} = 4, and Aged+MFG-E8 female: n_{mice} = 5). Quantitative analysis of the immunostaining intensities of SM-Myosin (h) (Aged male: n_{mice} = 3, Aged female: n_{mice} = 4, Aged+MFG-E8 male: n_{mice} = 5, and Aged+MFG-E8 female: n_{mice} = 4) and Runx2 (i) (Aged male: n_{mice} = 4, Aged female: n_{mice} = 5, Aged+MFG-E8 male: n_{mice} = 3, and Aged+MFG-E8 female: n_{mice} = 3) in the intimal medial area. Results are presented as mean ± SEM. Each data point was derived from an assessment of three sections from one individual animal. *$P < .05$, **$P < .01$, ***$P < .001$, and ****$P < .0001$, obtained using one-way analysis of variance followed by Tukey's multiple comparison test.

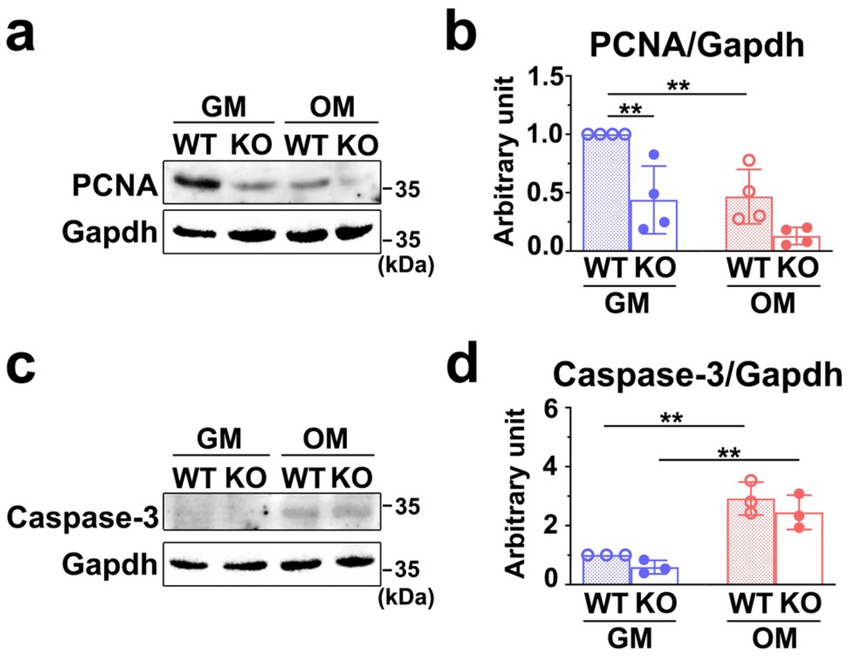

Supplementary Figure 2. Osteogenic medium (OM) does not alter the growth and apoptosis of primary vascular smooth muscle cells (VSMCs). VSMCs derived from the aortas of WT and MFG-E8-KO mice were cultured in growth medium (GM) and OM for 7 days. The protein expression of proliferating cell nuclear antigen (PCNA) (a) and caspase 3 (c) was evaluated through Western blotting. Quantitative analyses of PCNA (b) and caspase 3 (d) levels normalized to those of glyceraldehyde 3-phosphate dehydrogenase (GAPDH) were conducted (n = 3). Data are presented as the mean ± standard deviation. Three independent experiments were performed. Each data point is derived from each of the three repeated experiments. ***P* < .01, obtained using one-way analysis of variance followed by Tukey's multiple comparison test.

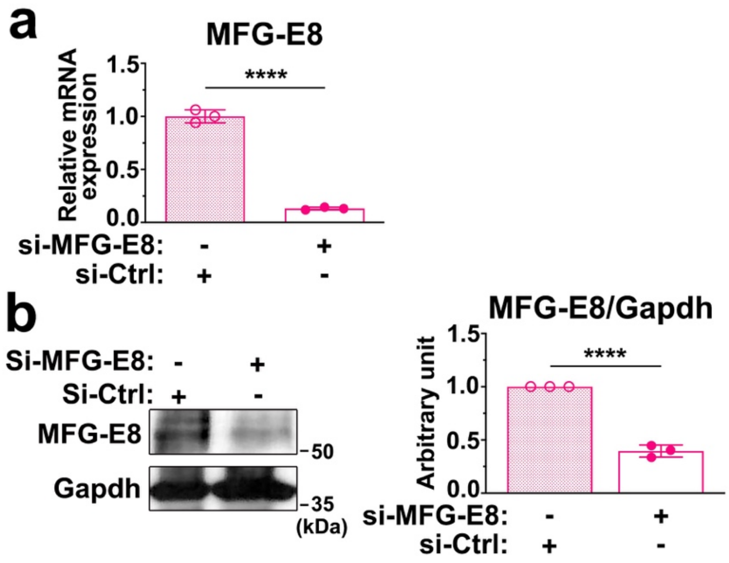

Supplementary Figure 3. Efficiency of the milk fat globule–epidermal growth factor 8 (MFG-E8) knockdown in A10 vascular smooth muscle cells (VSMCs). A10 VSMCs were transfected with siRNA against MFG-E8 (si-MFG-E8) or the control (si-Ctrl) for 48 h. (a) The transcript expression of MFG-E8 was evaluated through quantitative real-time polymerase chain reaction (n = 3). Data are presented as mean ± standard deviation (SD). Three independent experiments were performed, from which each data point was derived. ****$P < .0001$, as obtained using a t test. (b) Immunoblotting of the protein expression of MFG-E8 in A10 cells was performed. Quantitative analysis of MFG-E8 normalized to glyceraldehyde 3-phosphate dehydrogenase (GAPDH) was conducted (n = 3). Data are presented as mean ± SD. Three independent experiments were performed. Each point is derived from each of the three repeated experiments. ****$P < .0001$, as obtained using a t test.

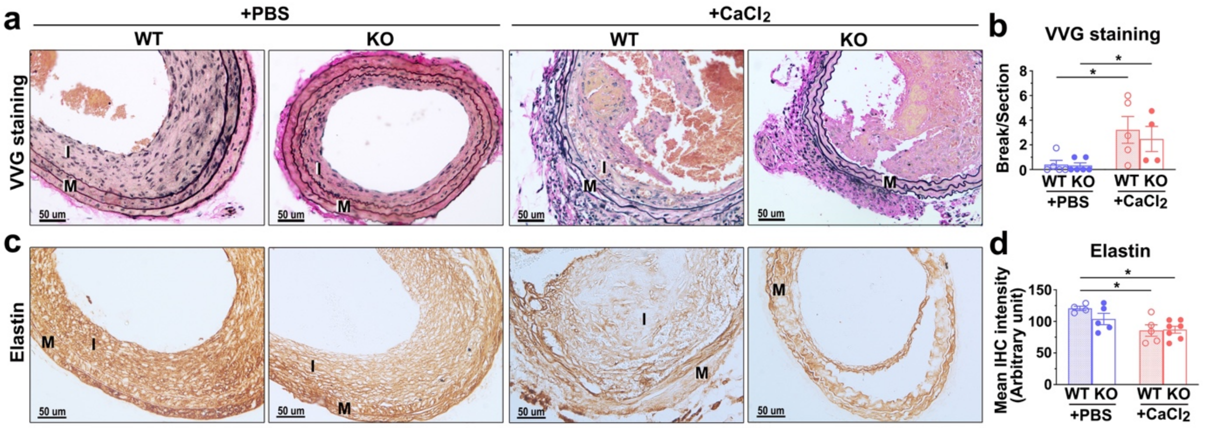

Supplementary Figure 4. Elastin fiber fragmentation and degradation is induced through $CaCl_2$ treatment both in wild-type (WT) and milk fat globule–epidermal growth factor (MFG-E8)-knockout (KO) mice. Ligated common carotid arteries (CCAs) of WT and MFG-E8-KO mice were treated with pluronic gel containing 0.4 M $CaCl_2$ to induce vascular calcification. (a) Representative images of Verhoeff–van Gieson (VVG) staining on CCA sections from four groups of mice 21 days postligation. Elastin breaks are indicated by arrowheads. Scale bar: 50 µm. The neointima (I) and media (M) of the vessels are indicated. (b) Quantification of number of elastin breaks per section (WT+phosphate-buffered saline (PBS): $n_{mice} = 5$, KO+PBS: $n_{mice} = 6$, WT+$CaCl_2$: $n_{mice} = 5$, KO+$CaCl_2$: $n_{mice} = 4$). Results are presented as mean ± standard error of the mean (SEM). Each point is derived from an assessment of 3–6 sections of an individual animal. *$P < .05$, as obtained using one-way analysis of variance (ANOVA) followed by Tukey's multiple comparisons test. (c) Representative immunohistochemistry (IHC) images of elastin in the CCAs of mice 21 days after ligation. Scale bar: 50 µm. The neointima (I) and media (M) of the vessels are indicated. (d) Quantitative analysis of the immunostaining intensities of elastin in the intimal medial area was performed (WT+PBS: $n_{mice} = 4$, KO+PBS: $n_{mice} = 5$, WT+$CaCl_2$: $n_{mice} = 5$, and KO+$CaCl_2$: $n_{mice} = 7$). Results are presented as the mean ± SEM. Each point was derived from an assessment of 3 sections of an individual animal. *$P < .05$, obtained using one-way ANOVA followed by Tukey's multiple comparison test.

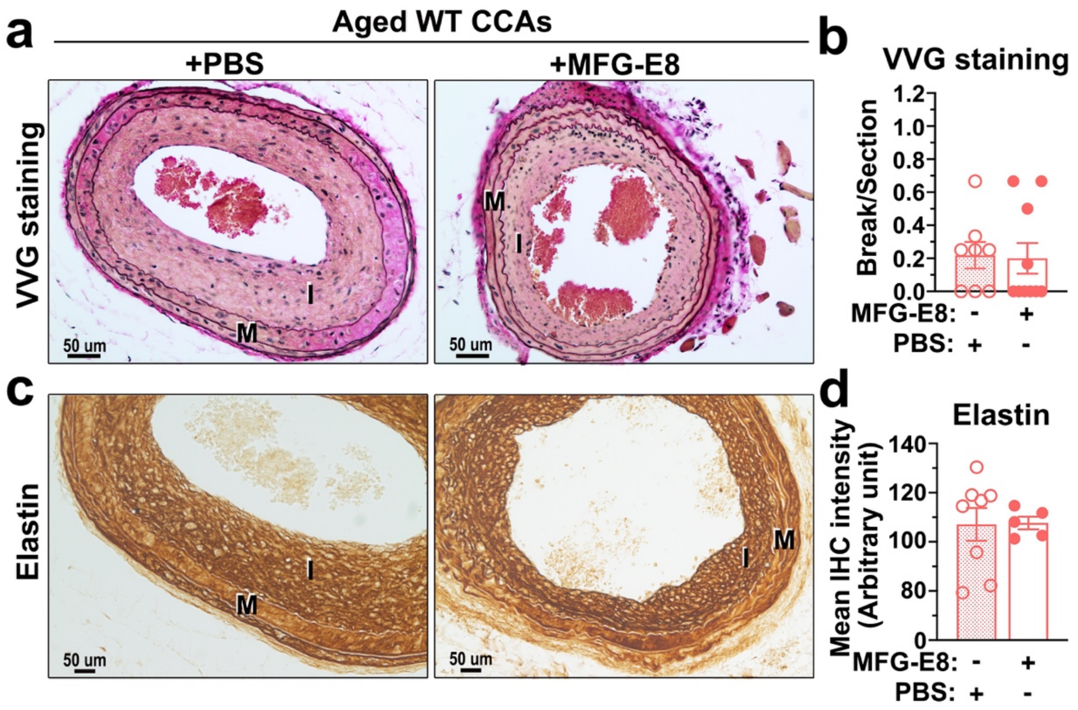

Supplementary Figure 5. Ligation injury on common carotid arteries (CCAs) of aged mice does not elicit significant elastin fiber fragmentation and elastin degradation in the vascular wall. The CCAs of aged wild-type (WT) mice were ligated to induce vascular remodeling. Pluronic gel containing phosphate-buffered saline (PBS) or rMFG-E8 was applied on CCAs immediately after the ligation. Paraffin sections of ligated CCAs 21 days postligation were subjected to Verhoeff–van Gieson (VVG) staining. The neointima (I) and media (M) of the vessels are indicated. (a) Representative images of CCA sections from two groups of mice. Scale bar: 50 μm. (b) Quantification of number of elastin breaks per section (WT+PBS: n_{mice} = 8, WT+MFG-E8: n_{mice} = 10). Results are presented as the mean ± standard error of the mean (SEM). Each point was derived from an assessment of 3 sections of one individual animal. (c) Representative IHC photomicrographs of elastin in the ligated CCAs 21 days after surgery. Scale bar: 50 μm. The neointima (I) and media (M) of the vessels are indicated. (d) Quantitative analysis of the immunostaining intensities of elastin in the intimal

medial area. (WT+ PBS: n_{mice} = 8, and WT+MFG-E8: n_{mice} = 5). Results are presented as the mean ± SEM.

Fig. 1d

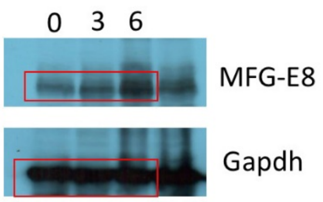

Supplementary Figure 6. Original images for immunoblots shown in Figure 1

Fig. 4e

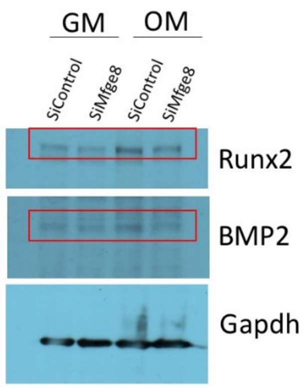

Supplementary Figure 7. Original images for immunoblots shown in Figure 4

Fig. 5b

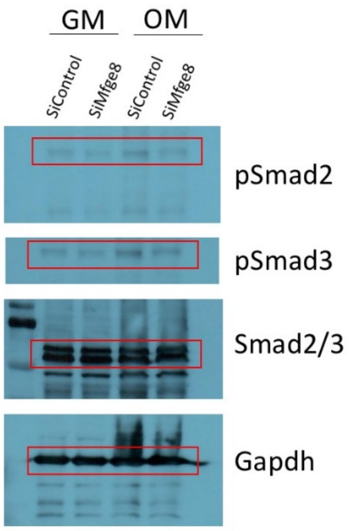

Supplementary Figure 8. Original images for immunoblots shown in Figure 5

Fig.6c

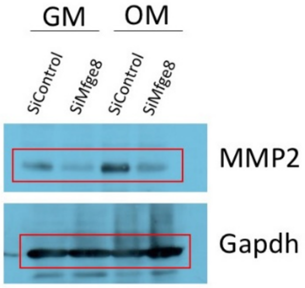

Fig.6h

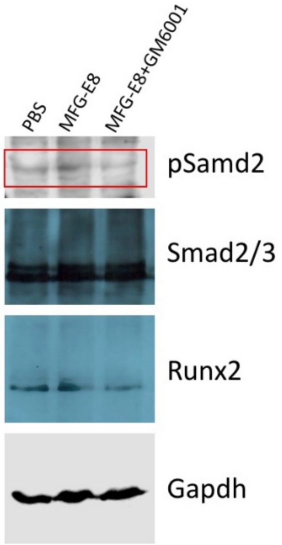

Fig.6k

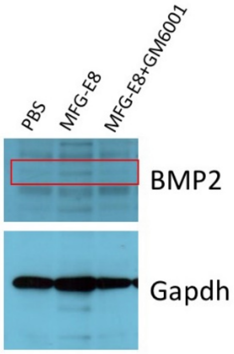

Supplementary Figure 9. Original images for immunoblots shown in Figure 6

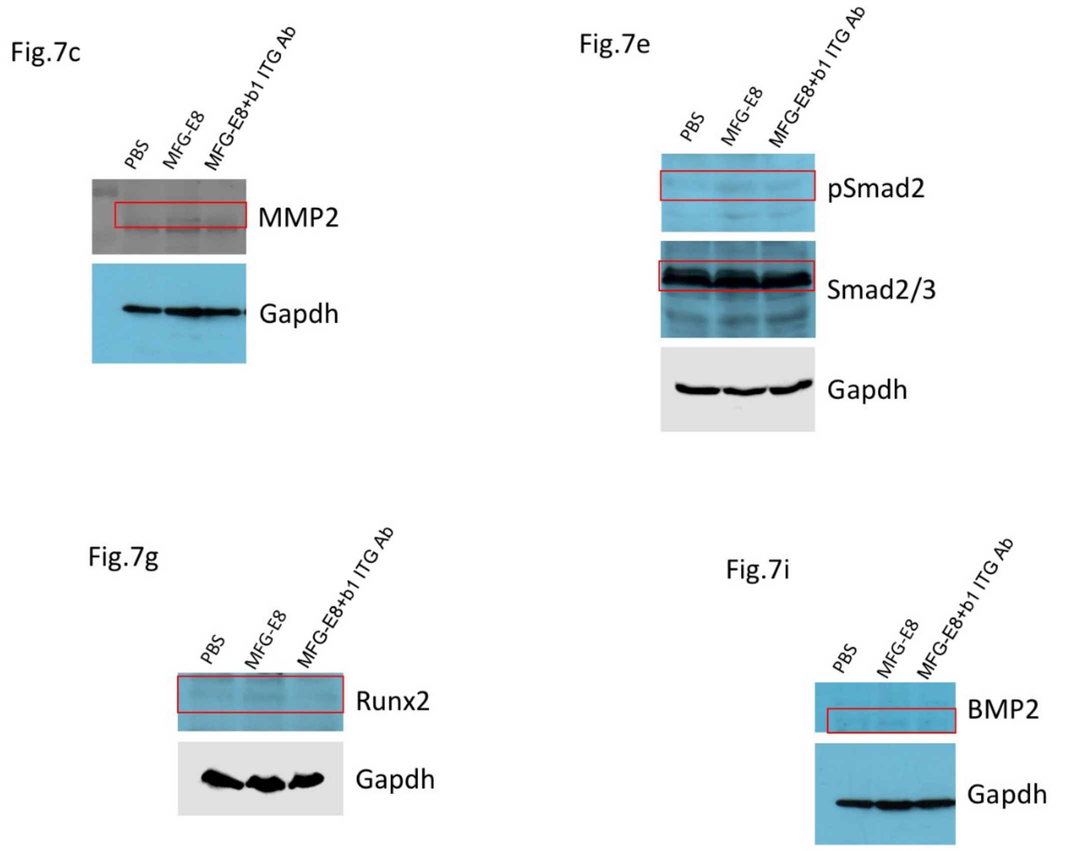

Supplementary Figure 10. Original images for immunoblots shown in Figure 7

Supplementary Tables

Supplementary Table I. Oligonucleotide sequences for quantitative real-time PCR.

Primer	Forward	Reversed
**Rat SMA**	5'-TGCTGCTTCCTCTTCTTC-3'	5'-GGTCCTTCCTGATGTCAATA-3'
**Rat SM-Myosin**	5'-CGATGAGGTGGTTGTAGA-3'	5'-CCGAGTAGGTGTAGATGAG-3'
**Rat PAI-1**	5'-CGTGCCTTGCTCTTTATC-3'	5'-CTCCTTAATAGTGCTTCTTCTC-3'
**Rat MMP2**	5'-ATCAGCCTTCTCCTTCAC-3'	5'-GTCAGCACCTTTCTTTGG-3'
**Rat MMP9**	5'-TGCTCCAACTGCTGTATA-3'	5'-GTGTCCTCCGATGTAAGA-3'
