# Supplementary material for: MFG-E8 promotes osteogenic transdifferentiation of smooth muscle cells and vascular calcification by regulating TGF-β1 signaling
Source: Commun Biol. 2022 Apr 19;5:364. doi: 10.1038/s42003-022-03313-z (PMC9018696; doi:10.1038/s42003-022-03313-z)
Supplement: Supplementary file 2 — Description of Additional Supplementary Files [file 42003_2022_3313_MOESM2_ESM.pdf]

# Description of Additional Supplementary Files

**File name:** Supplementary Data 1
**Description:** Source data underlying the graphs.
