# Supplementary material for: MFG-E8 promotes osteogenic transdifferentiation of smooth muscle cells and vascular calcification by regulating TGF-β1 signaling
Source: Commun Biol. 2022 Apr 19;5:364. doi: 10.1038/s42003-022-03313-z (PMC9018696; doi:10.1038/s42003-022-03313-z)
Supplement: Supplementary file 4 — Reporting Summary [file 42003_2022_3313_MOESM4_ESM.pdf]

nature research

Corresponding author(s): Ting-Hein Lee

Last updated by author(s): Mar 17, 2022

Reporting Summary

Nature Research wishes to improve the reproducibility of the work that we publish. This form provides structure for consistency and transparency in reporting. For further information on Nature Research policies, see our Editorial Policies and the Editorial Policy Checklist.

Statistics

For all statistical analyses, confirm that the following items are present in the figure legend, table legend, main text, or Methods section.

n/a	Confirmed	
☐	☒	The exact sample size (*n*) for each experimental group/condition, given as a discrete number and unit of measurement
☐	☒	A statement on whether measurements were taken from distinct samples or whether the same sample was measured repeatedly
☐	☒	The statistical test(s) used AND whether they are one- or two-sided<br>*Only common tests should be described solely by name; describe more complex techniques in the Methods section.*
☐	☒	A description of all covariates tested
☐	☒	A description of any assumptions or corrections, such as tests of normality and adjustment for multiple comparisons
☐	☒	A full description of the statistical parameters including central tendency (e.g. means) or other basic estimates (e.g. regression coefficient) AND variation (e.g. standard deviation) or associated estimates of uncertainty (e.g. confidence intervals)
☐	☒	For null hypothesis testing, the test statistic (e.g. *F*, *t*, *r*) with confidence intervals, effect sizes, degrees of freedom and *P* value noted<br>*Give P values as exact values whenever suitable.*
☒	☐	For Bayesian analysis, information on the choice of priors and Markov chain Monte Carlo settings
☒	☐	For hierarchical and complex designs, identification of the appropriate level for tests and full reporting of outcomes
☒	☐	Estimates of effect sizes (e.g. Cohen's *d*, Pearson's *r*), indicating how they were calculated

Our web collection on statistics for biologists contains articles on many of the points above.

Software and code

Policy information about availability of computer code

Data collection: N/A

Data analysis: N/A

For manuscripts utilizing custom algorithms or software that are central to the research but not yet described in published literature, software must be made available to editors and reviewers. We strongly encourage code deposition in a community repository (e.g. GitHub). See the Nature Research guidelines for submitting code & software for further information.

Data

Policy information about availability of data

All manuscripts must include a data availability statement. This statement should provide the following information, where applicable:

- Accession codes, unique identifiers, or web links for publicly available datasets
- A list of figures that have associated raw data
- A description of any restrictions on data availability

N/A

Field-specific reporting

Please select the one below that is the best fit for your research. If you are not sure, read the appropriate sections before making your selection.

☒ Life sciences ☐ Behavioural & social sciences ☐ Ecological, evolutionary & environmental sciences

For a reference copy of the document with all sections, see nature.com/documents/nr-reporting-summary-flat.pdf

Life sciences study design

All studies must disclose on these points even when the disclosure is negative.

Sample size	Based on our experiences and other published reports, 3-10 mice/group is sufficient to show the phenotype of each experiment group.
Data exclusions	There are no data excluded.
Replication	All attempts at replication were successful.
Randomization	Mice subjected to each experimental procedures were randomly chosen.
Blinding	The investigators were blind in analyzing data.

Reporting for specific materials, systems and methods

We require information from authors about some types of materials, experimental systems and methods used in many studies. Here, indicate whether each material, system or method listed is relevant to your study. If you are not sure if a list item applies to your research, read the appropriate section before selecting a response.

Materials & experimental systems

n/a	Involved in the study	
☐	☒	Antibodies
☐	☒	Eukaryotic cell lines
☒	☐	Palaeontology and archaeology
☐	☒	Animals and other organisms
☒	☐	Human research participants
☒	☐	Clinical data
☒	☐	Dual use research of concern

Methods

n/a	Involved in the study	
☒	☐	ChIP-seq
☒	☐	Flow cytometry
☒	☐	MRI-based neuroimaging

Antibodies

Antibodies used	anti-smooth muscle myosin antibody (Biomedical Technologies, BT562), anti-Runx2 antibody (Cell Signaling, #12556), anti-activated β1 integrin antibody (Merck Millipore, MAB2079Z), anti-MFG-E8 antibody (R&D, AF2805), anti-phosphorylated Smad2 antibody (Cell signaling, #3108), anti-phosphorylated Smad3 antibody (Cell signaling, #9520), anti-Smad2/3 antibody (Cell signaling, #8685), anti-BMP2 antibody (Genetex, GTX14933), anti-elastin antibody (Santa Cruz, SC-166543)
Validation	The validation statements of all antibodies used are available on the manufacturer's website.

Eukaryotic cell lines

Policy information about cell lines

Cell line source(s)	A10 cells were acquired from Bioresource Collection and Research Center (Hsinchu, Taiwan).
Authentication	This cell line is commercial available.
Mycoplasma contamination	The cell line is not tested for mycoplasma contamination.
Commonly misidentified lines (See ICLAC register)	*Name any commonly misidentified cell lines used in the study and provide a rationale for their use.*

Animals and other organisms

Policy information about studies involving animals; ARRIVE guidelines recommended for reporting animal research

Laboratory animals	Male and female WT and MFG-E8-KO mice (6–8 weeks old) backcrossed to the FVB/NJNarl background were used in this study.
Wild animals	This study did not involve wild animals.
Field-collected samples	This study did not involve samples collected from the field.
Ethics oversight	All animal procedures were approved by the Institutional Animal Care and Use Committee of Chang Gung University

Note that full information on the approval of the study protocol must also be provided in the manuscript.
